# *The Whole Is Greater than the Sum of Its Parts* – Challenges and Perspectives in Polyelectrolytes

**DOI:** 10.1021/acs.biomac.4c01061

**Published:** 2024-12-11

**Authors:** Anja Traeger, Meike N. Leiske

**Affiliations:** a Institute of Organic Chemistry and Macromolecular Chemistry (IOMC), Friedrich Schiller University Jena, 07743 Jena, Germany; b Jena Center for Soft Matter (JCSM), Friedrich Schiller University Jena, 07743 Jena, Germany; c Macromolecular Chemistry, University of Bayreuth, 95447 Bayreuth, Germany; d Bavarian Polymer Institute, 95447 Bayreuth, Germany

## Abstract

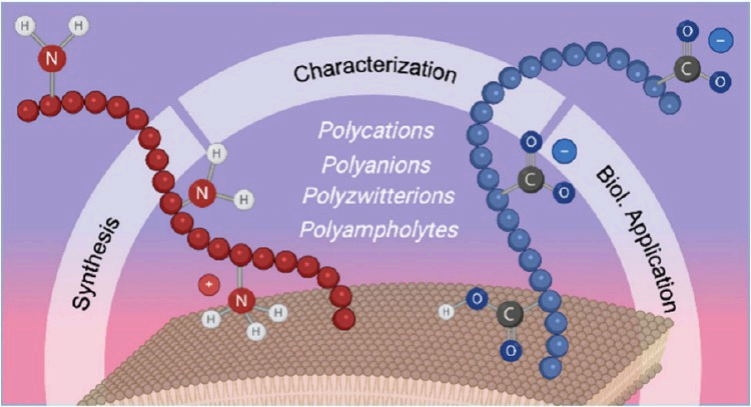

Polyelectrolytes offer unique properties for biological applications due to their charged nature and high water solubility. Here, the challenges in their synthesis and characterization techniques are reviewed, emphasizing that their strong interactions with the surrounding media and counterions must be considered when working with this interesting class of materials. Their potential in complexation for gene delivery, their unique stealth and anti-fouling properties, and their more specific interactions with amino acid transporters for cancer therapy are highlighted. The underlying mechanisms responsible for their biological efficacy, including the proton sponge effect for endosomal release and their interactions with cellular membranes, are addressed. For polyelectrolytes with a high level of usage, an overview is given of their historical context. This Perspective outlines the potential of polyelectrolytes for innovative applications in the field of biomedicine. Considering the physicochemical characteristics of this class of materials, this work strives to elucidate the distinctive properties and applications of polyelectrolytes.

## 1 Introduction

The insight by Aristotle, “The whole is greater than the sum of its parts,” is more appropriate in the case of polyelectrolytes than for polymers in general, due to their high charge density and complex interactions with their surroundings. Polycations, polyanions, polyzwitterions, and polyampholytes exhibit unique properties that arise due to the interplay of their charged components.^1^ Their performance, influenced by electrical forces and interactions with biological systems, leads to a myriad of functional possibilities that are also known from some natural-derived counterparts (proteins, polysaccharides).^2^ They have great potential for biological applications due to their versatility, chemical flexibility, and multifunctionality.^3^ In medicine, they are being explored for drug delivery, tissue engineering and diagnostics, offering solutions for controlled release, targeted delivery, and improved therapeutic efficacy.^4^ A special class of charged polymers, polyelectrolytes, have proven to be of particular interest in the fight against cancer.^5^ Their unique ability to interact with biological molecules and cell membranes to form more complex structures enables the development of new therapeutic approaches. Recent research developments and the continuous lack of tissue-specific medications for cancer treatment led to an urgent need for further exploration of these treatments to not only improve the therapeutic success but also reduce the severe side effects in patients.^6^

Modern nanomedicine focuses on the development of carrier systems for active pharmaceutical ingredients (APIs; herein referred to as “drugs”) to improve their site-specificity.^7,8^ The use of polymers is rapidly emerging as a promising avenue in drug delivery. The conjugation of drugs to polymers or their physical entrapment can improve solubility,^9^ blood circulation time,^10^ and tissue distribution.^11^ This can lead to a reduction of interactions with non-tumor cells and a minimization of side effects such as hair loss, cardiotoxicity, and anemia.^6^ To this end, drug carriers can be composed of lipids (e.g., liposomes or (solid) lipid nanoparticles),^12^ biomacromolecules (e.g., protein-derived carriers),^13^ or synthetic polymers (e.g., nanoparticles, polymersomes, or polymer–drug conjugates).^14^ Loading active small-molecule drugs into these nanocarriers typically increases their water solubility and, consequently, enhances their bioavailability and biodistribution. Meanwhile, the risk of non-specific tissue interactions is mitigated.^14,15^ While structural modifications of the actual drugs are limited to not compromising their therapeutic efficiency, drug delivery carriers can be tailored regarding their size, shape, stiffness, hydrophilicity, surface chemistry, or stimuli responsiveness.^16,17^

To avoid unwanted immune reactions and side effects, materials often include non-ionic, low-fouling moieties, such as polyethylene glycol (PEG), which enables an accumulation of nanocarriers in cancerous tissue through exploitation of the enhanced retention permeation (EPR) effect.^18^ Further, its modulation of the protein’s corona actively avoids clearance.^19^ Stealth or low-fouling compounds, introduced into the carrier’s outer layer or surfaces, reduce its interaction with biological matter (i.e., proteins and cell surfaces) and consequently increase the blood circulation time drastically.^20^ Due to the leaky structure of the tissue around cancer cells, nanomaterials possess an increased chance to diffuse into tumors rather than into healthy tissues.^21^ While stealth/low-fouling properties are advantageous to decrease unwanted cellular interactions, they can also reduce the material’s association with the target tissue and cells, also known as the PEG dilemma.^22,23^ Although this technique has the potential to enhance the site-specific delivery of drugs to a limited extent,^24^ it is not as effective as active targeting strategies, which seek to bind nanocarriers with distinctive structures on the cell surface.^25^ To this end, polymers functionalized with biological motifs, such as sugars, antibodies, or amino acids, have shown great potential to accumulate in tumors and cancer cells.^26−29^

The diverse applications and interactions of polyelectrolytes create a vital area of research, with significant implications for the advancement of nanomedicine. The potential of polycations for gene and drug delivery facilitates cellular uptake, membrane crossing, and protection of labile drugs. In contrast, polyanions and polyzwitterions can interact with amino acid transporters (AATs) in a selective manner, which enables targeted delivery and uptake. Besides, the stealth properties of polyelectrolytes enable their application in systemic circulation and targeted drug delivery, and their anti-fouling properties, achieved through surface modification, prevent the formation of unwanted biofilms and protein adsorption, ensuring the efficiency and longevity of biomedical devices. This Perspective addresses the synthesis of polyelectrolytes, presenting a variety of methodologies that facilitate precise control over the molar mass, charge density, and functional group distribution. It also addresses the inherent difficulties in the field of analytics, including the characterization of intricate architectures and dynamic behaviors. This overview further highlights the impact of polyelectrolytes in nanomedicine while also providing a critical assessment of their synthesis and analytical challenges inherent to the field.

## 2 Polyelectrolytes – General Considerations

**Figure 1:**
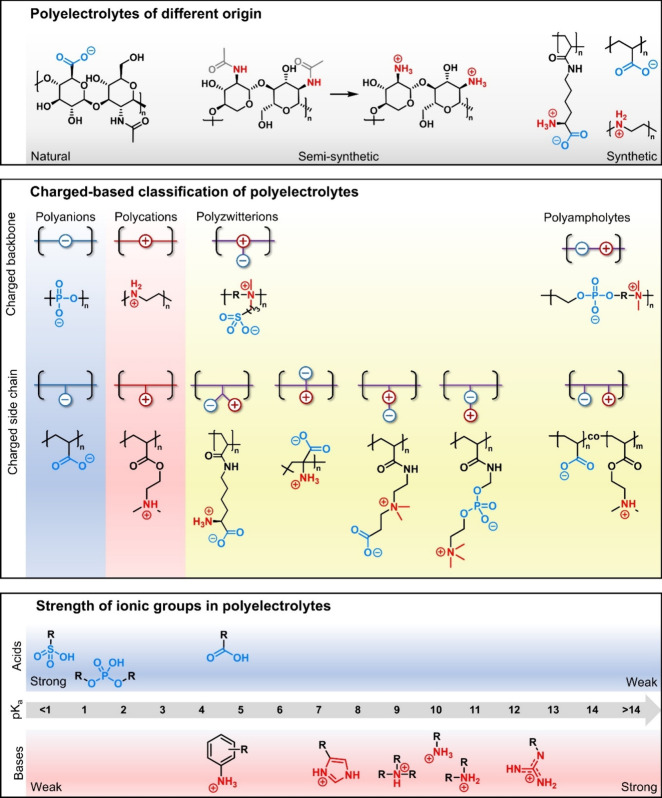
Schematic structures and examples of different polyelectrolytes and representative chemical structures of exemplary polymers. Cationic charges are highlighted in red and anionic ones are highlighted in blue. Please note that the apparent p*K*_a_ value of a polyelectrolyte depends on multiple factors, including the residue R. For simplicity, here R is assumed to be an alkyl group.

According to the IUPAC nomenclature, a polyelectrolyte is defined as a “polymer composed of macromolecules in which a substantial portion of the constitutional units contains ionic or ionizable groups, or both.” ^30^ For simplicity, this Perspective will use the term “ionic groups” only. In general, polyelectrolytes can be classified into four different categories: (i) polyanions, which contain anionic groups; (ii) polycations, which contain cationic groups; (iii) polyampholytes, which contain both anionic and cationic groups; and (iv) polyzwitterions, a unique type of polyampholyte in which anionic and cationic groups are present in each monomer unit (Figure 1).

In general, polyelectrolytes can be either of (semi)synthetic or of natural origin. Examples of natural polyelectrolytes are polysaccharides,^31^ gelatin,^32^ proteins,^33^ and (deoxy)ribonucleic acid (DNA/RNA),^34^ while semisynthetic polyelectrolytes are often based on polysaccharides or alginates.^35^ The ionic groups may be located in the polymer backbone, side chain, or both. While polyanions and polycations commonly feature only one type of ionic group, polyampholytes and zwitterions are more complex,^36,37^ the latter also known as zwitterionic polymers (ZIPs), polymeric inner salts, or polybetaines.^38,39^

In the following sections, we will provide an overview of their general properties (e.g., hydration and solubility), characterization methods, and synthetic approaches yielding different polyelectrolytes. The emphasis will be on polymer types that are relevant for biomedical applications, as a later part of this Perspective will focus on their interactions in biological environments.

## 3 Synthesis of Polyelectrolytes

In the past, polyelectrolytes have been synthesized in numerous different ways. Modifications of naturally occurring polymers such as saccharides or chitosan allow for the preparation of semisynthetic polyelectrolytes.^40^ For example, chitin may serve as a starting material for the synthesis of chitosan polyelectrolytes via deacetylation.^41^ Post-polymerization modification (PPM) of synthetic polymers has also emerged as a promising strategy to synthesize polyelectrolytes which may not or hardly be accessible via conventional polymerization techniques.^42^ Polymerization techniques that allow for using aqueous conditions as reaction solvent (e.g., free radical polymerization (FRP)) are compatible with the polymerization of unprotected monomers with ionizable side chains. Examples include the synthesis of poly(acrylic acid) (PAA)^43^ and the polymerization of sulfobetaine monomers,^44^ among others.

The synthesis of polyelectrolytes—in particular via controlled or living techniques—may face some obstacles along the way. The reasons for these challenges are derived from their structure and properties: (i) high hydrophilicity and (ii) nucleophilic functional groups. The exceptional hydrophilicity of polyelectrolytes leads to a limited solubility in very hydrophilic (often protic) solvents,^45,46^ which may be incompatible with a wide range of polymerization techniques, including ionic polymerizations. In addition, the nucleophilicity of the functional groups or the structural motifs themselves may interfere with further established methods, such as reversible deactivation radical polymerizations (RDRPs). Reversible addition–fragmentation chain transfer (RAFT) polymerization is sensitive to the use of primary amines due to aminolysis of the chain-transfer agent (CTA), while atom-transfer radical polymerization (ATRP) is incompatible with carboxylic acids, as they are known to complex the copper catalyst. Strategies for the syntheses of polyelectrolytes have been reviewed elsewhere.^46−48^

PPM has emerged as a feasible strategy to obtain polyelectrolytes while circumventing the issues described above. Here, ionic moieties may be introduced into polymer chains using straightforward exchange techniques requiring activated monomers.^42,49−51^ Orthogonally reactive moieties can be applied to yield polyelectrolytes even in the absence of protecting groups. In addition, polybetaines may be synthesized via PPM of poly(2-(dimethylamino)ethyl dimethacrylate) (PDMAEMA; section 3.2.4), circumventing solubility issues of (poly)zwitterions during the polymerization itself.

Furthermore, protecting groups have been shown to be useful for the preparation of monomeric and polymeric precursors, which only reveal their ionic character upon deprotection.^48^ Besides protecting the functional groups from unwanted side reactions, these groups also increase the hydrophobicity of the compounds and consequently increase their solubility in organic, non-protic solvents, such as chloroform, dimethylformamide, or even toluene.

In the following section, we highlight different approaches that were established for the synthesis of different kinds of polyelectrolytes: (i) polycations, (ii) polyanions, (iii) polyzwitterions, and (iv) polyampholytes (Figure 1).

### 3.1 General Considerations

While incredibly interesting for applications in the biomedical research area, the functional groups of polyelectrolytes are disadvantageous for the preparation of polymers due to their interference with the polymerization techniques or decreased solubility in commonly applied organic solvents. In the following paragraphs we aim to provide a short overview of these limitations as well as potential solutions that have been reported in the literature. We first focus on polycations and then polyanions. Polyzwitterions and polyampholytes will not be discussed in detail in the general section, as they combine the challenges of polycations and polyanions.

**Figure 2:**
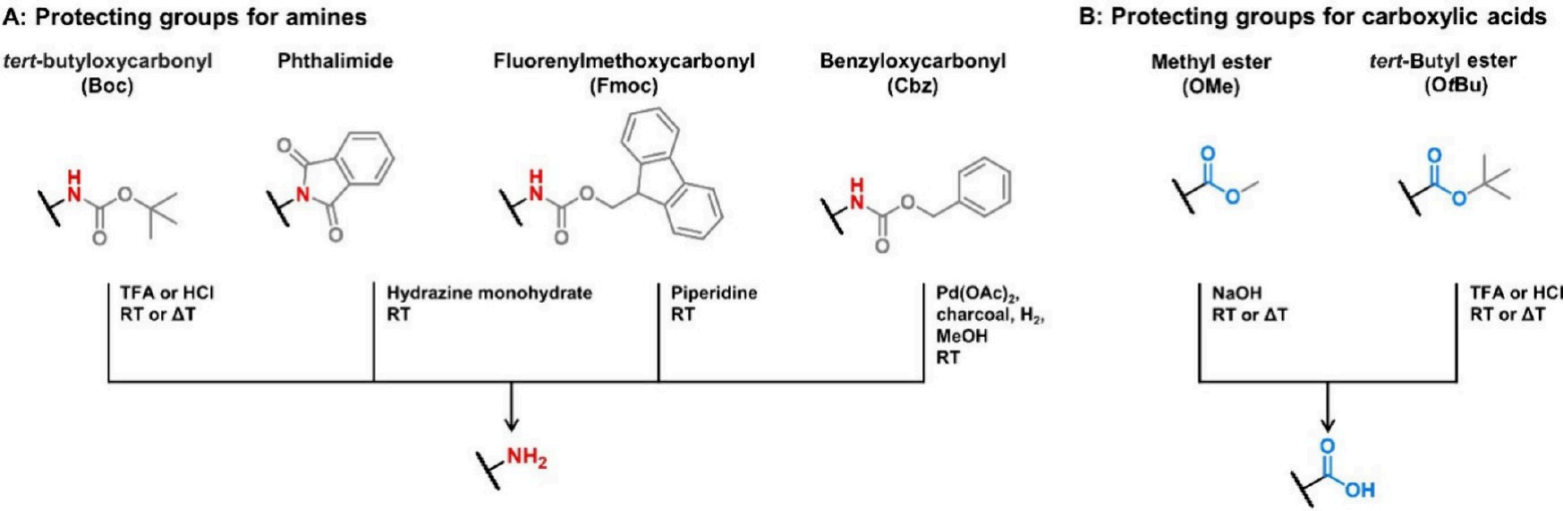
Commonly applied protecting groups (gray) for (A) amines or (B) carboxylic acids and their respective deprotection conditions.

The preparation of polycations—in particular featuring amino groups—via ionic polymerization techniques requires the use of protecting groups (Figure 2) to mask those potentially interfering moieties.^52^ The same applies for polyanions, which feature strongly nucleophilic acid groups (e.g., carboxylates). In contrast to ionic polymerizations, radical polymerization techniques feature a higher tolerance toward functional groups.^53^ For example, monomers with pendant tertiary amines are perfectly suitable for radical polymerizations. In contrast, primary and secondary amines need to be protected with protecting groups as described above to enable RAFT polymerization, which is sensitive to aminolysis of the CTA.^53^ Particular care should be taken with regard to the choice of protecting unit (Figure 2), as acid deprotection is favored over basic deprotection, which may—as a side reaction—lead to the degradation of other moieties, such as esters in poly(meth)acrylates. In contrast, FRP and ATRP are compatible with free amines.

Polycations are polymers with a significant fraction of positive charges. These charges are most often derived from amino groups. Unfortunately, they interfere with a variety of polymerization techniques, e.g., ionic polymerizations or RAFT polymerization, and hence protecting groups are required.^54^ Commonly applied protecting groups for primary and secondary amines are *tert*-butyloxycarbonyl (Boc), phthalimides, fluorenylmethoxycarbonyl (Fmoc), or benzyloxycarbonyl (Cbz) groups.^55,56^ Here, the choice of protecting group ultimately relies on the suitability of the protectant with the monomer synthesis and polymerization conditions. For example, the Fmoc protecting group is unlikely to be suitable for a monomer synthesis that also requires the use of a base such as piperidine. Another important factor is the inertness of the resulting polymer structure toward the applied deprotection conditions. Polymers with heteroatoms such as ester or amide groups may suffer from unwanted degradation.

### 3.2 Polycations

It is nearly impossible to imagine research in polymer nanomedicine without polycations. Their positive ionic charge renders them attractive candidates for applications including, but not limited to, gene transfer or polymeric antimicrobials.^54^ In the following paragraphs, we address the synthetic strategies of a few prominent polycations in more detail.

#### 3.2.1 Polyalkyleneimines

**Figure 3:**
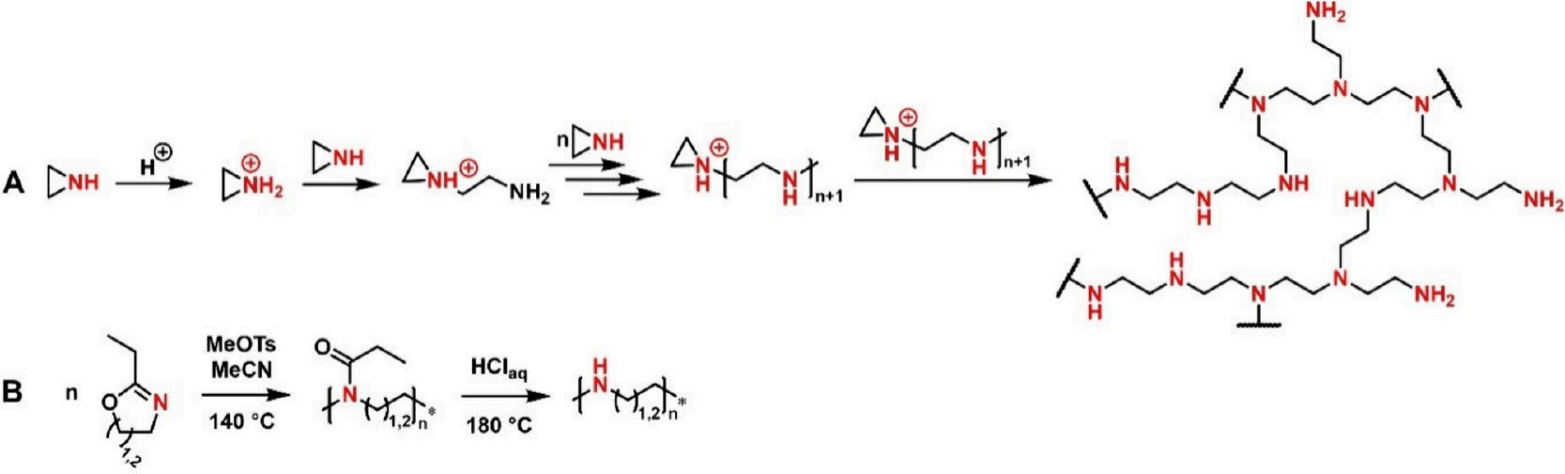
Synthesis of polyethyleneimines: (A) branched polyethyleneimine amd (B) linear polyethyleneimine.

For decades, **polyethyleneimine (PEI)** was considered the gold-standard for polymeric gene transfection. PEI’s development was driven by the need for materials with unique charge properties and versatile chemical functionalities. There are two architectures of PEI available, branched (*b*-PEI) and linear (*l*-PEI), which are synthesized via two fundamentally different processes (Figure 3).^57^*b*-PEI is synthesized via the cationic ring-opening polymerization (CROP) of aziridine, which is initiated via an electrophile (e.g., the proton from a protic acid). Protonation of the nitrogen leads to a positive charge and consequently favors the attack of an additional aziridine unit onto the ring of the protonated species, thus leading to a ring-opening reaction. These reactions will continue in chain-growth polymerization. However, the reaction of two ring-opened species is also possible, leading to branched polymers which contain a mixture of primary, secondary, and tertiary amines.^58^

In contrast, *l*-PEI is synthesized in a two-step process. First, a 2-oxazoline monomer (e.g., 2-ethyl-2-oxazoline) is polymerized via living CROP, yielding a linear poly-2-oxazoline.^52^ In a second reaction, this polymeric precursor is then hydrolyzed under strongly acidic (or basic) conditions, yielding secondary amines located in the polymeric backbone.^59^ Recently, another approach was established in which a protected aziridine derivative is polymerized via anionic ring-opening polymerization, yielding linear polymers. In a second step a deprotection is necessary to yield the *l*-PEI derivative featuring methyl groups branching off the backbone.^57^ At this point it is worth mentioning that the use of azetidines or 2-oxazines yields **linear polypropyleneimines (*l*-PPIs)** instead.^57,60^ These are, compared to PEIs, far less explored; however, they have shown promise in the context of polymeric gene transfection.^60,61^

**Poly(β-amino esters) (PBAEs)**, polycations which feature main-chain amines as well as degradable ester groups, are synthesized via a stepwise approach. The starting compounds for these polymers are (di)amines and diacrylates, which react in a one-pot Michael addition. Variations in the resulting structures are plentiful, as the variety of amines and acrylates that may be used is immense.^62^ Compared to *l*-PEIs or *l*-PPIs, which are synthesized via CROP, PBAEs feature a higher dispersity; however, their degradability renders them interesting for applications in nanomedicine such as gene delivery.^63^

#### 3.2.2 Dendritic Polycations

In contrast to linear polymers, branched or dendritic polymers feature a more spherical shape and, consequently, a high density of functional surface groups. As described above, *b*-PEI is synthesized via CROP of aziridine. **Branched polypropyleneimine (*b*-PPI)**, however, must be synthesized in a stepwise manner.^64^ In this divergent synthesis approach, a mono- or diamine serves as the starting point for the synthesis of the first generation. Acrylonitrile reacts with the amino groups in a cyanomethylation reaction, which must be catalyzed by an acid (e.g., acetic acid). It was furthermore reported that the reaction solvent is of critical importance for the success of the reaction. In particular, heterogeneous systems such as emulsions of water and toluene were found suitable in the past. After the cyanomethylation, the nitrile group is reduced to an amino group via reductive hydrogenation under high pressure using, for example, Raney cobalt as catalyst.^65^

Another prominent dendritic polycation, **poly(amidoamine) (PAMAM)**, is synthesized in a similar stepwise manner. Three different approaches were established in the past: (i) divergent synthesis, (ii) convergent synthesis, and (iii) combined divergent/convergent synthesis.^66^ The divergent synthesis is similar to the synthesis of *b*-PPI, as the dendrimer is constructed from a core, growing outward.^67^ In contrast, in convergent synthesis the outer dendritic branches are built first before being attached to the core.^68^ These approaches are generally tedious, as they require the use of protecting groups and multiple purification steps. In the case of PAMAM, primary amines are reacted with methyl acrylate in a Michael-type addition.^69,70^ Subsequently, the methoxy groups are reacted with excessive 1,2-diethylamine in transamidation reactions. Iterative cycles of these reactions finally result in dendritic structures. Combined approaches attach smaller dendritic building blocks to already extended cores, aiming for accelerated synthesis with reduced defects.^71^ The resulting dendrimers additionally often feature the potential for higher generations.

#### 3.2.3 Amino-Acid-Derived Polycations

Amino acids are naturally occurring zwitterions which feature an amine and a carboxylic acid group in the α-position. This high degree of functionality renders them interesting tools for organic synthesis. Consequently, they have been employed in numerous reactions, yielding amino-acid-derived polymers which are often of cationic character.^48,72^**Poly(α-**l-lysine) (PLL) is a synthetic biopolymer that finds applications in the biomedical field. Its isomer, **poly(ε-**l-lysine), is a naturally occurring biopolymer,^73^ which can also be prepared via biosynthesis in organisms such as *Streptomyces albulus*.^74^ Prominent examples of the applications of PLL include polymeric antimicrobials, gene delivery, drug delivery, protein delivery, and tissue engineering.^75^ It is commonly synthesized via three different routes: (i) solid-phase peptide synthesis (SPPS), (ii) ring-opening polymerization (ROP), or (iii) chemo-enzymatic synthesis. The choice of technique is generally motivated by the requirements of the product, as they yield PLL of different molar mass (distribution) and purity.

**Figure 4:**
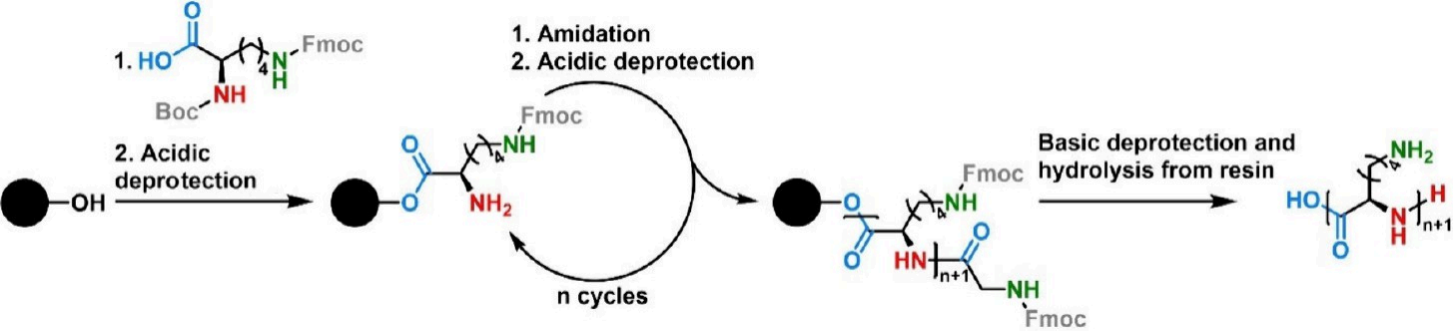
Schematic simplified depiction of the SPPS of a Lys derivative, yielding PLL.

SPPS is a stepwise approach that allows for the synthesis of short polypeptides (<50 amino acids).^76,77^ In general, an *N*-terminal protected amino acid is covalently attached to a solid support (i.e., a resin) and then deprotected. After that, a second, likewise *N*-terminal protected amino acid is reacted with the first amino acid. The deprotection–amidation cycle is repeated until the desired peptide structure is obtained (Figure 4). In the synthesis of PLL via this method, two orthogonal protecting groups (e.g., Boc and Fmoc, Figure 2) are required. The main advantage of this method is the potential to create sequence-defined polypeptides of different amino acids. However, a drawback is the limited molar mass that can be reached. As sequence definition is not required for these homopolymers, ROP came to the forefront as an alternative method for the preparation for PLL which is now the most prevalently used.

When applying ROP, PLL is synthesized via the polymerization of α-amino acid *N*-carboxyanhydrides (NCAs). As a living polymerization technique, ROP yields a PLL in high yield and with narrow dispersity. Due to the sensitivity of both ROP and NCAs against weak bases (i.e., water, alcohols, and amines), the ε-amino group requires protection prior to the NCA synthesis. The polymerization mechanism of amino-acid-derived NCAs has been comprehensively reviewed recently.^48^

Chemo-enzymatic synthesis makes use of enzymes as biocatalysts. It has been successfully applied to the synthesis of different polypeptides, including PLL, under relatively mild conditions. While this method is a cost-effective and environmentally friendly alternative to SPPS and ROP, it often suffers from a low molar mass of the resulting polymers.

Amino-acid-derived poly(meth)acrylates have been reported in the past.^48^ These polymers are often synthesized via RAFT polymerization of Boc-protected monomers and subsequently deprotected to yield polymers with cationic side chains.^72,78^ The use of different amino acids enables the preparation of polycations with tailored hydrophobicity.^79^ Polycations with guanidine moieties in the side chain were previously synthesized in a similar manner.^80^

#### 3.2.4 Vinylic Polymers Featuring Tertiary Amines

**Figure 5:**
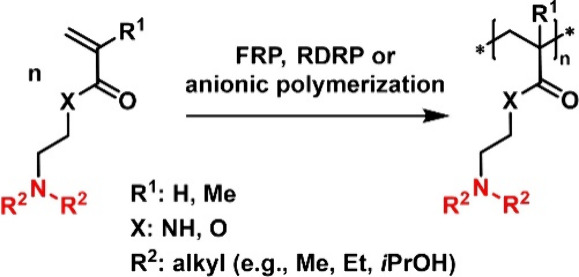
Synthesis of vinylic polymers with tertiary amines in the side chain via free radical polymerization (FRP), reversible deactivation radical polymerization (RDRP), or anionic polymerization. Charges are omitted for simplicity.

In recent years, **PDMAEMA** and other polymethacrylates featuring tertiary amines (e.g., poly(diisopropylaminoethyl dimethacrylate)) have gained attention due to their responsiveness to pH and temperature and their potential to act as gene delivery vectors, facilitating endosomal release while maintaining considerably high biocompatibility.^81^ These polymers are generally prepared in a straightforward manner via radical polymerization techniques (Figure 5), as they do not require protecting groups. Most often, the polymers are synthesized via ATRP or RAFT polymerization;^81^ however, synthesis via anionic polymerization^82,83^ was also established in the past. PDMAEMA might be the most commonly prepared polymer of this kind, but **poly(2-(dimethylamino)ethyl acrylate) (PDMAEA)** gained attention due to its self-degrading properties^84^ and was consequently synthesized with increasing interest in the past. The synthesis of (meth)acrylamide-derived polymers with tertiary amines in the side chain has been less explored to date.^85^ In addition to linear polymers, the use of these RDRP techniques enables the preparation of polymer architectures such as branched star-shaped polymers with cationic arms.^86−88^

### 3.3 Polyanions

Although they receive less attention in macromolecular science, it is hard to imagine modern medicine without the use of polyanions. Like polycations, they find applications in many different areas, such as coatings, vaccine additives, and antimicrobials. In the following paragraphs, an overview of the synthesis of different polyanions featuring carboxylates as anionic moieties is provided.

#### 3.3.1 Amino-Acid-Derived Polyanions

**Poly(glutamic acid) (PGA)**, which finds applications in the biomedical area, can be produced in two different constitutions, namely, as the α- and γ-isomers. They find applications as antimicrobials, in drug delivery, and in tissue engineering.^89^ The γ-isomer of PGA is produced via biosynthesis from microorganisms (e.g., *Bacillus* strains).^90^ In contrast, the α-isomer of PGA can be synthesized by ROP of glutamic-acid-derived NCAs.^91^ Hereby, the γ-carboxylic acid group must first be protected before the NCA is synthesized by refluxing the precursor with phosgene. The ROP is initiated via standard conditions,^48^ enabling the preparation of high-molar-mass PGA after deprotection of the γ-carboxylic acid. In the past, PGA or the related poly(aspartic acid) has furthermore served as a precursor for the preparation of polycations, where the resulting free carboxylic acid was reacted with 1,2-diaminoethane in an amidation reaction to yield the desired compounds.^92^

#### 3.3.2 PAA and Related Polymers

**PAA**—potentially the most abundant polyanion—finds applications in numerous fields, ranging from life science (e.g., drug delivery)^93^ to the personal care sector (e.g., superabsorbents).^94^ As a vinyl-derived polymer, it is usually prepared by radical polymerization techniques, e.g., FRP or RDRP. Using a water-soluble initiator such as 4,4′-azobis(4-cyanopentanoic acid) (ACPA), PAA can be prepared by FRP in aqueous media.^95^ If narrower molar mass distributions are required, acrylic acid can also be polymerized via different RDRP techniques. In this context, nitroxide-mediated polymerization (NMP) was found to be unsuitable due to nitroxide’s decomposition in acidic media.^96^ RAFT polymerization, which is compatible with carboxylates and acidic conditions, was successfully applied as the polymerization method for PAA.^97,98^ Due to issues with complexation with the copper catalyst, polymerization of acrylic acid via ATRP remains difficult and commonly yields only polymers with poor dispersity.^99^ Polymerization of precursor, *tert*-butyl acrylate, with subsequent acidic hydrolysis represents one alternative option to circumvent this issue.^100^ In addition to enabling ATRP, the use of *tert*-butyl acrylate offers the additional advantage of organo-solubility, which, consequently, enables anionic polymerization^101^ as well copolymerization with a wide range of functional hydrophobic monomers,^102^ yielding polyanions with tailored properties.

**Figure 6:**
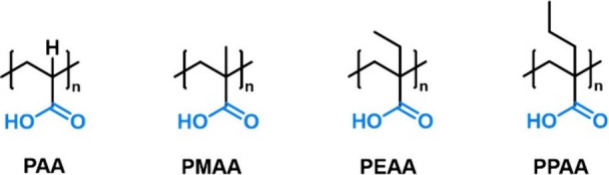
Chemical structures of poly(acrylic acid) (PAA), poly(methacrylic acid) (PMAA), poly(ethylacrylic acid) (PEAA), and poly(propylacrylic acid) (PPAA). Charges are omitted for simplicity.

While copolymerization represents a straightforward strategy to obtain polyanions with tailored hydrophobicity and charge density, in recent years the development of different polyanions has been of great interest. One reason for this interest is the simplification of the system to be applied to biological environments to enable more precise conclusions on structure–property relationships.^42^ PAA derivatives with increased hydrophobicity through backbone substituents may be synthesized via radical polymerizations of methyl,^103^ ethyl,^104^ or propyl^105^ acrylates (Figure 6). However, it should be pointed out here that the polymerizability of ethyl and propyl acrylates is generally decreased compared to that of acrylates and methacrylates. In the 1950s, copolymers of PMAA and poly(methyl methacrylate) were launched under the name Eudragit.^106,107^

#### 3.3.3 Polyacrylamides

**Figure 7:**
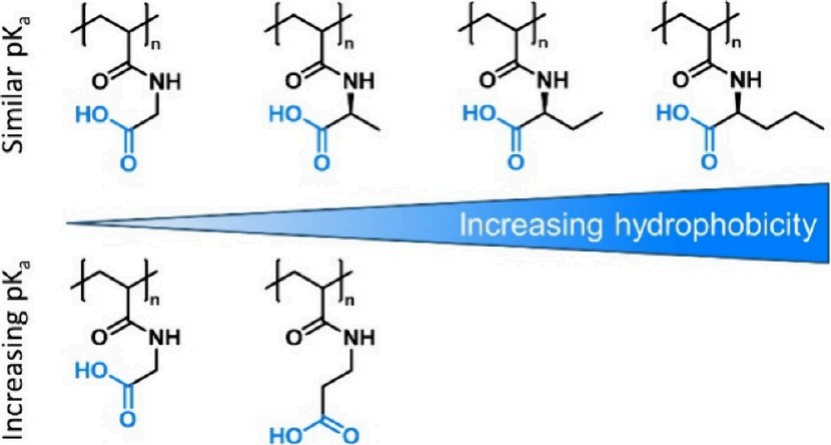
Schematic representation of carboxylated polyacrylamides with tailored hydrophobicity and p*K*_a_ values.^42,109^ Charges are omitted for simplicity.

In addition to modifications of the polymer backbones, previously the development of polyacrylamide equivalents came to the forefront (Figure 7). One strategy covers the synthesis of carboxylate acrylamide monomers with distinct alkyl spacers between the acrylamide and the carboxylate (i.e., methyl or ethyl).^108,109^ Compared with PAA, these polymers possess easily tunable p*K*_a_ values combined with varying hydrophobicity. Another approach which has been explored recently is the synthesis of amino-acid-derived polycarboxylates via polymer-analogous reactions. Precisely, this strategy uses activated ester precursors, which are reacted in a post-polymerization amidation suspension with amino acids.^42^ Compared with the functional monomer approach, this approach allows for the avoidance of tedious monomer synthesis and purification protocols. In addition, highly comparable polymers can be prepared from the same precursor if the reaction conditions are suitable for anhydrous reactions, which enable quantitative modifications and minimize unwanted hydrolytic byproducts. The resulting polymers feature comparable p*K*_a_ values combined with a systematic increase in hydrophobicity.

### 3.4 Polyzwitterions

In recent years, polyzwitterions have drawn attention for use in the biomedical field.^110,111^ Applications of these polymers include drug delivery, anti-fouling coatings, stabilizers for nanoparticles and proteins, and self-healing hydrogels.^112^ In contrast to polyelectrolytes, which are classified into polycations and polyanions, polyzwitterions contain oppositely charged cationic and anionic groups along the chain or side chain of one macromolecule.^46^ To this end, the charge stoichiometry of the resulting polymers can be equal or an excess of one type of charge. Besides polyzwitterions, polyampholytes contain opposite charges on separate repeating units distributed throughout the chain.^113^ Biomacromolecules such as proteins or peptides are polyampholytes with a sequence-defined pattern in their primary structure, which impacts their 3D structure. Synthetic polyampholytes may be synthesized in an alternating, statistical (i.e., random, or gradient-like), or block-like manner. In contrast to polyampholytes, polyzwitterions contain zwitterionic moieties within one repeating unit (RU). Zwitterionic RUs appear to be more similar to naturally occurring (neutral) amino acids. Therefore, in the following paragraphs, emphasis will be placed on the synthesis and properties of polymers with zwitterionic RUs.

**Figure 8:**
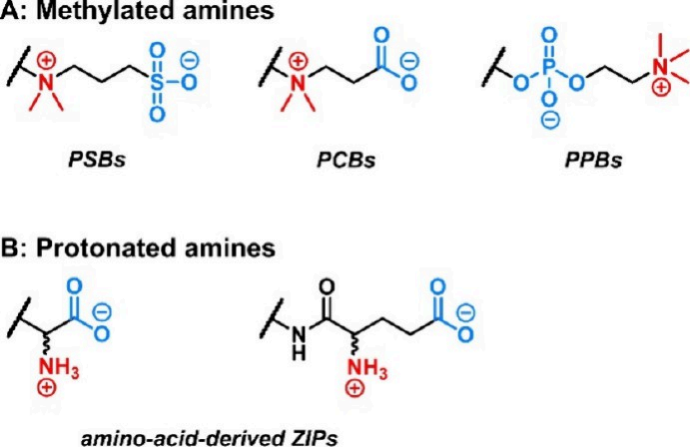
Structures of zwitterionic polymer side chains: (A) zwitterions with methylated amines and (B) zwitterions with protonated amines.

The majority of compounds that have been studied in biological environments in this context are polysulfobetaines (PSBs), polycarboxybetaines (PCBs), and polyphosphobetaines (PPBs), which is why ZIPs are often referred to as polybetaines.^112^ Hereby, charges can be introduced in an irreversible manner (Figure 8A), e.g., via methylation,^47^ or reversibly (Figure 8B), e.g., through protonation.^114^ The anionic charge can be provided through different functionalities, such as carboxylates, sulfates, phosphates, or acylsulfonamides. Due to the polar character of these highly charged molecules, solvents for polymerizations are often limited to water or other very hydrophilic solvents.^45−47^ Thus, the preparation of polymers with zwitterionic RUs is commonly mediated through radical polymerizations, as they are inert to a broad variety of solvents, including water. In the following paragraphs, the synthesis of (protected) zwitterionic vinyl monomers will be presented in order to facilitate a more profound comprehension of the synthetic prerequisites that must be considered as well as to give insights into the challenges and benefits of the diverse range of zwitterionic monomers.

#### 3.4.1 Synthesis of Polybetaines

Polybetaines can be designed by either preparation of functional monomers or PPM of respective polymers.^45−47^ The different types of polybetaines as well as their synthesis and characterization have been summarized in great detail before.^45^ In the following paragraphs, the synthesis of polymeric betaines is exemplarily described for examples with biological relevance. To this end, two different synthetic routes have been widely explored: (i) polymerization of betaines and (ii) PPM.^46^

While the polymerization of betaines may lead to difficulties caused by incompatibilities with a range of polymerization techniques, poor solubility of intermediates and reaction products, and, consequently, challenging characterization, the use of neutral or protected precursors has revealed itself to be a helpful tool to minimize these issues. Polymers comprising tertiary amines in the side chain can easily be quarternized while installing the anionic moiety in the same step.^46,112^ In general, these approaches are compatible with a variety of polymerization techniques; however, radical polymerization is the most widely explored and will therefore be discussed for PSBs and PCBs in more detail in the following paragraphs, as these are the polybetaines that are most commonly applied at the biointerface.

##### PSBs

**Figure 9:**
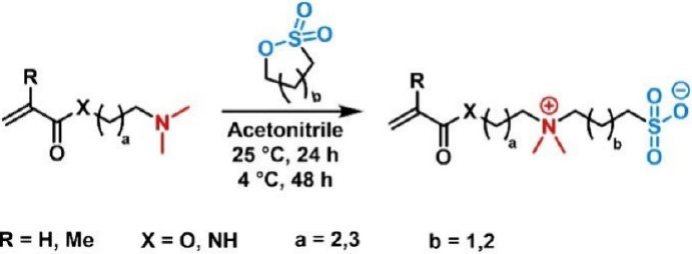
Reaction of dimethylamino-substituted vinyl monomers with sultones, yielding sulfobetaines.^116^

The synthesis of PSBs is facilitated by different strategies, but the reaction of a tertiary amine (e.g., DMAEMA) with a sultone (i.e., 1,3-propanesultone or 1,4-butanesultone) is the most common (Figure 9).^115^ In this reaction, the sultone acts as an alkylsulfonating agent. The reaction proceeds in acetonitrile at ambient conditions and was reported to yield the desired polymerizable vinylic sulfobetaines in quantitative yields without the need for elaborate purification techniques.^116^ Alternative reactions are possible using haloalkylsulfonates instead of sultones. Radical polymerization of these vinylic monomers in aqueous media yields PSBs.^117,118^

##### PCBs

**Figure 10:**
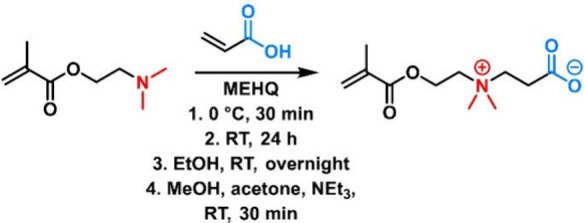
Reaction of dimethylamino-substituted vinyl monomers with acrylic acid, yielding carboxybetaines.^115^

The synthesis of PCBs is facilitated by different routes. A common procedure features the synthesis of a tertiary amine (e.g., DMAEMA) with an α,β-unsaturated acid, such as acrylic acid (Figure 10). In a typical procedure, DMAEMA and acrylic acid are mixed in bulk in the presence of a radical inhibitor and stirred at 0 °C for 30 min, followed by another 4 h at room temperature. Upon increasing viscosity of the reaction mixture, a dilution with ethanol is performed and then the mixture is stirred further overnight. Subsequently, the desired product can be isolated by the addition of methanol, acetone, and triethylamine, which leads to precipitation within 30 min.^115^ In alternative synthetic routes, tertiary amines are reacted with haloalkylcarboxylates^119^ or haloalkylcarboxylic esters.^47^ Polymerization of these vinylic monomers can be conducted in a manner similar to that described above for sulfobetaines.

#### 3.4.2 Amino-Acid-Derived Polymers

While polybetaines have been researched intensively in recent years, polyzwitterions comprising α-amino acids in the side chain have only recently started to draw attention as alternative zwitterionic materials.^114,120−122^ Polyzwitterions can be synthesized from proteinogenic amino acids. To this end, acidic amino acids (e.g., glutamic acid or aspartic acid) or basic amino acids (i.e., lysine (Lys)) can serve as the starting material for monomer preparation. To avoid non-specific reactions during the monomer synthesis, it is recommended to use amino acids which comprise protected amino and carboxylic acid groups. The introduction of protecting groups can also be beneficial for the subsequent purification and polymerization of the obtained monomers due to their more hydrophobic character. Commonly used protecting groups are the Boc group for amines and the *tert*-butyl (*t*Bu) group for carboxylic acids, which can both be deprotected quantitatively under acidic conditions, such as treatment with trifluoroacetic acid.

**Figure 11:**
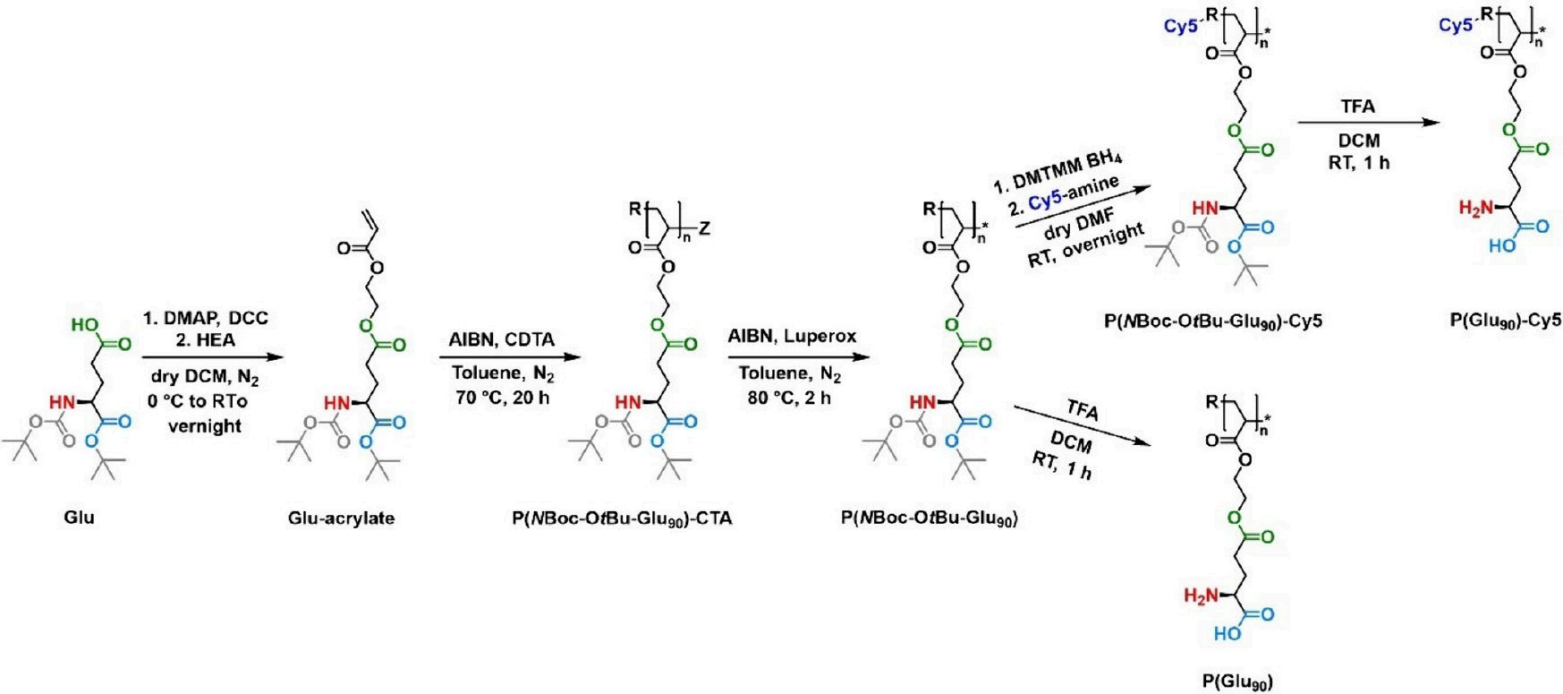
Synthesis of zwitterionic Glu-derived polyacrylates from NBoc-OtBu-Glu. Adapted with permission from ref (114). Copyright 2022 American Chemical Society.

In a recent example, *N*Boc-Glu-O*t*Bu was reacted with 2-hydroxyethylacrylate in a straightforward Steglich esterification, which was conducted with the aid of DMAP and DCC (Figure 11).^114,122^ The desired product, *N*Boc-O*t*Bu-Glu acrylate, was yielded with high purity. Notably, this synthetic method also allowed for the preparation of *N*Boc-O*t*Bu-Glu acrylamide and *N*Boc-O*t*Bu-Glu methacrylate monomers. In contrast, *N*Boc-O*t*Bu-Glu methacrylamide was prepared via the reaction of *N*Boc-Glu-O*t*Bu with 2-isopropenyl-2-oxazoline. Polymers of all four different vinyl monomers were synthesized via RAFT polymerization, allowing the introduction of a fluorescent label (i.e., Cy5 or fluoresceine) at the R end group prior to deprotection.

**Figure 12:**
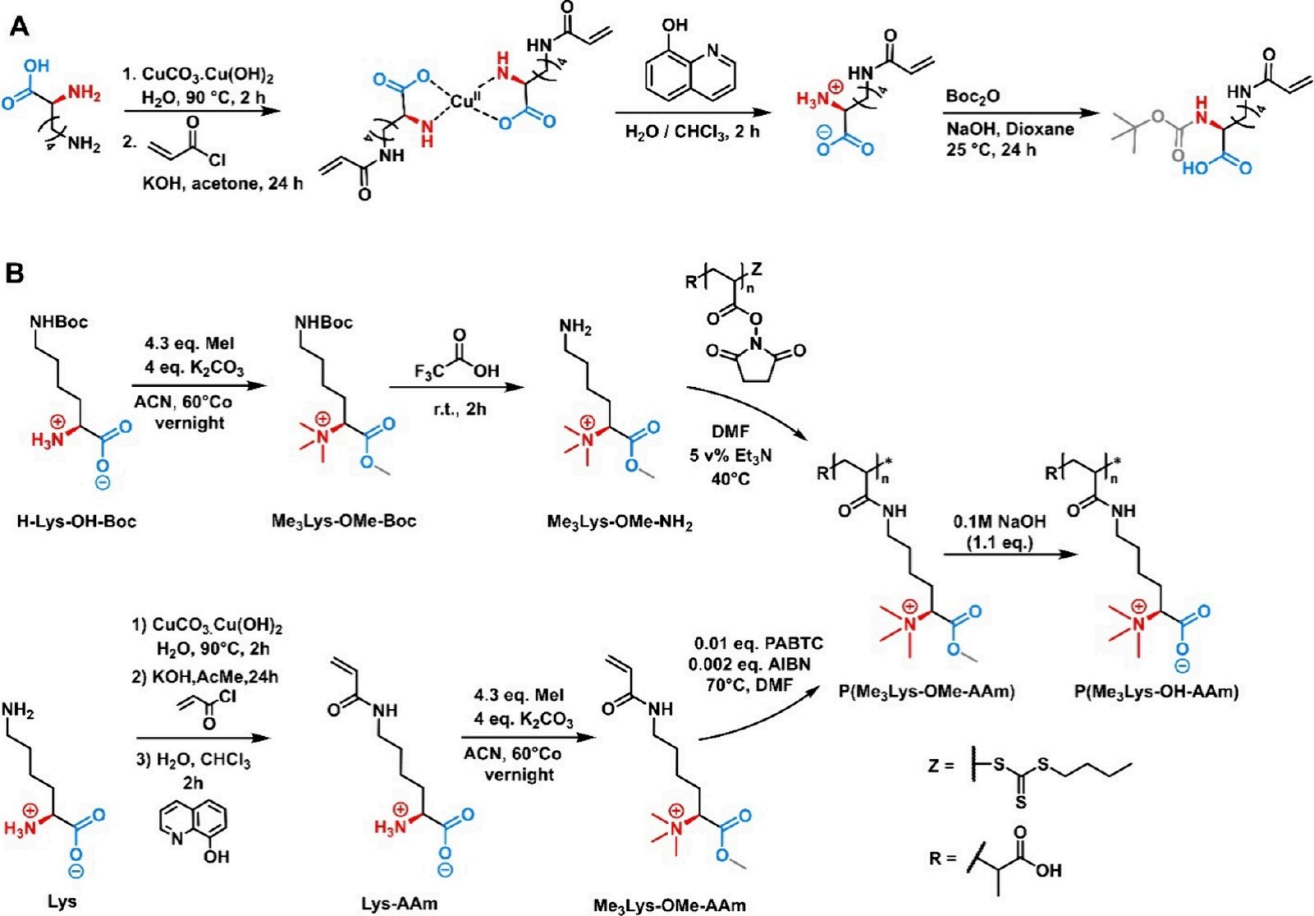
Synthesis of Lys-derived polyacrylamides for the preparation of zwitterionic polymers. Panel A adapted with permission from ref (123). Copyright 2022 American Chemical Society. Panel B adapted with permission under a Creative Commons CC_BY 4.0 license from ref (49). Published 2024 Wiley.

In another, very elegant approach, P. Dinda et al. synthesized the l-Lys-derived acrylamide monomer Boc-Lys-acrylamide from unprotected l-Lys (Figure 12A).^123^ In a first reaction, the amino acid was complexed with basic copper carbonate, leading to a quasi-protection of the α-amino acid group. In a subsequent reaction, they reacted the δ-amino group with acryloyl chloride, thus forming the acrylamide moiety. After decomplexation of the copper carbonate with 8-hydroxyquinoline, the resulting Lys-acrylamide was treated with di-*tert*-butyl dicarbonate, yielding the desired compound Boc-Lys-acrylamide, readily available for RAFT polymerization. Recently, De Breuck et al. applied this synthetic strategy to synthesize chiral, amino-acid-derived polybetaines from l-Lys (Figure 12B).^49^ In addition to the RAFT polymerization of the functional monomer, the potential to prepare these polymers via PPM was successfully explored.

Poly(dehydroalanine), an additional polymer that is less explored in the biological area, is also synthesized via radical polymerization (i.e., ATRP) of a protected vinylic precursor, which is subsequently deprotected, resulting in a polyzwitterion that bears opposite charges at distinct backbone positions of the identical repeating unit.^124,125^

## 4 Key Properties and Characterization of Polyelectrolytes

### 4.1 A Short Introduction to Polyelectrolyte Theory

Knowledge of polyelectrolytes is important not only for the application of synthetic polymers in nanomedicine but also for a better understanding of important biopolymers. The best-known representatives of biological polyanions are certainly the polynucleic acids (DNA and RNA), along with structural proteins such as pectic in fruit jellies or heparin. The theory behind the properties of polyelectrolytes is complex and has been described in the past.^126^ Within this section, we aim to provide a short overview of the most important methods relevant for biological applications. Within the past decades, the understanding of polymer properties in general has improved significantly with the development of analytical methods.^127^ Despite synthesis-derived uncertainties such as exact molar mass or composition of materials, non-ionic systems are quite well understood. However, the introduction of ionic charges into macromolecules adds another layer of complexity to the system. In particular, the effects of charge interactions—be they attractive or repulsive—as well as counterions or solvent salinity on the polymer structure complicate direct experimental tests and in-depth understanding. For this reason, simulations are of great importance for understanding polyelectrolytes; however, the interactions with the surrounding media require more complex and thus time-consuming methods.

### 4.2 Impact of Hydration, Solubility, and Counterions

Due to their ionic nature, polyelectrolytes are generally highly soluble in polar solvents such as water. During the solvation process, they dissociate into charged macro-ions and small counterions. To understand this process, one needs to consider different interactions occurring at the same time: (i) interactions between charged groups, (ii) hydrophobic forces, and (iii) hydrogen bonding.^128^

**Figure 13:**
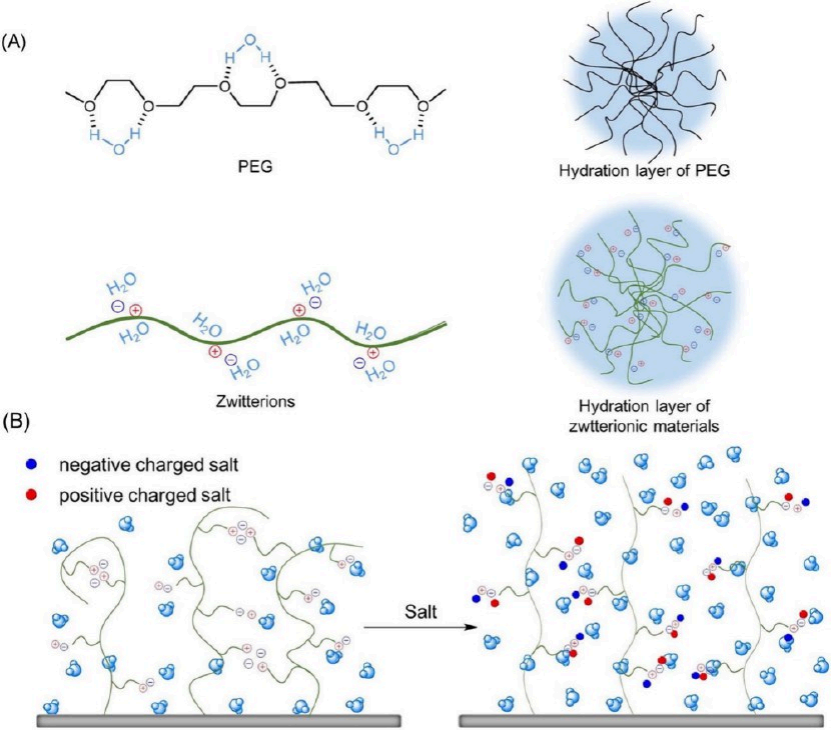
Schematic diagram of the hydration shell formed by PEG and zwitterions separately. (A) PEG interacts with H_2_O molecules via hydrogen bonding, while zwitterions attract H_2_O molecules through powerful ion–dipole interactions, forming stronger hydration layers to prevent bioadherence. (B) Incorporation of salts disrupts the previous electrostatic attraction of the intramonomer, intrachain, and interchain charge pairs, making the conformation of zwitterionic brushes shrink and stretch. Reproduced with permission from ref (129). Copyright 2020 Elsevier.

Under ideal conditions, the hydration of polyelectrolytes is significantly higher compared to that of non-ionic hydrophilic polymers (e.g., PEG), allowing for better solvation in aqueous media. The main driving force of the hydration of non-ionic polymers is hydrogen bonding, while polyelectrolytes form stronger hydration layers via dipole–dipole interactions (Figure 13A). However, the hydration is furthermore dependent on the counterions or the salinity of the solution (Figure 13B). The principles influencing the hydration of polyelectrolytes are summarized in the following paragraphs.

**Figure 14:**
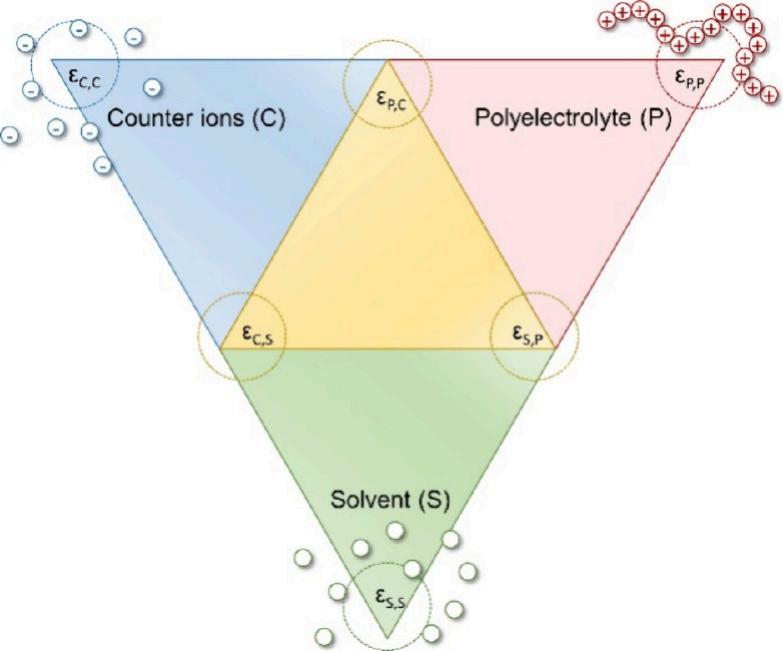
Schematic illustration of different energetic interactions among three species in polyelectrolyte solutions: polyelectrolytes, counterions, and solvent. The scheme exemplarily shows polycations.

To gain a deeper understanding of the properties of polyelectrolytes, it is essential to consider the factors that govern the solvation of simple ions such as inorganic salts. It is of paramount importance to account for the interactions between the various components, including the polyelectrolyte, counterions, and the solvent (Figure 14). In this context, the interactions within each species as well as the interactions of the different species with each other are important. Weak polyelectrolytes may cluster in solution.^130^ It was furthermore shown that the solvation of the polyelectrolyte depends on the solubility of the counterion;^131^ however, it may also be enhanced by strong interactions of the polyelectrolyte with the solvent.^132^ For example, Vlachy and colleagues showed that, due to their more hydrophobic character, bromide anions feature a higher affinity to ionene moieties units than fluoride anions.^133^ The decreased solubility of polyelectrolytes that have a strong affinity to their counterions is driven by decreased hydration of the ionic groups and, consequently, lower solvation in hydrophilic solvents.^134^ Although this has been studied for model systems, it is almost impossible to extend conclusions from these results to additional systems, leading to tedious procedures and simulation approaches. Here, machine-learning (ML) approaches could be of particular interest to predict the solvation of ionic (co)polymers and its effects on membrane interactions.

The size or accessibility of the ionic group is, furthermore, of central importance for its solubility. As an easy example, the solubility of ammonium ions decreases in order as NH_4_^+^ > RNH_3_^+^ > R_2_NH_2_^+^ > R_3_NH^+^ > R_4_N^+^ (where R is a simple alkyl residue such as a methyl group) due to the increased radius of the charge center.^135^ In a similar manner, charge delocalization (e.g., in carboxylates) leads to decreased solvation.^136^

**Figure 15:**
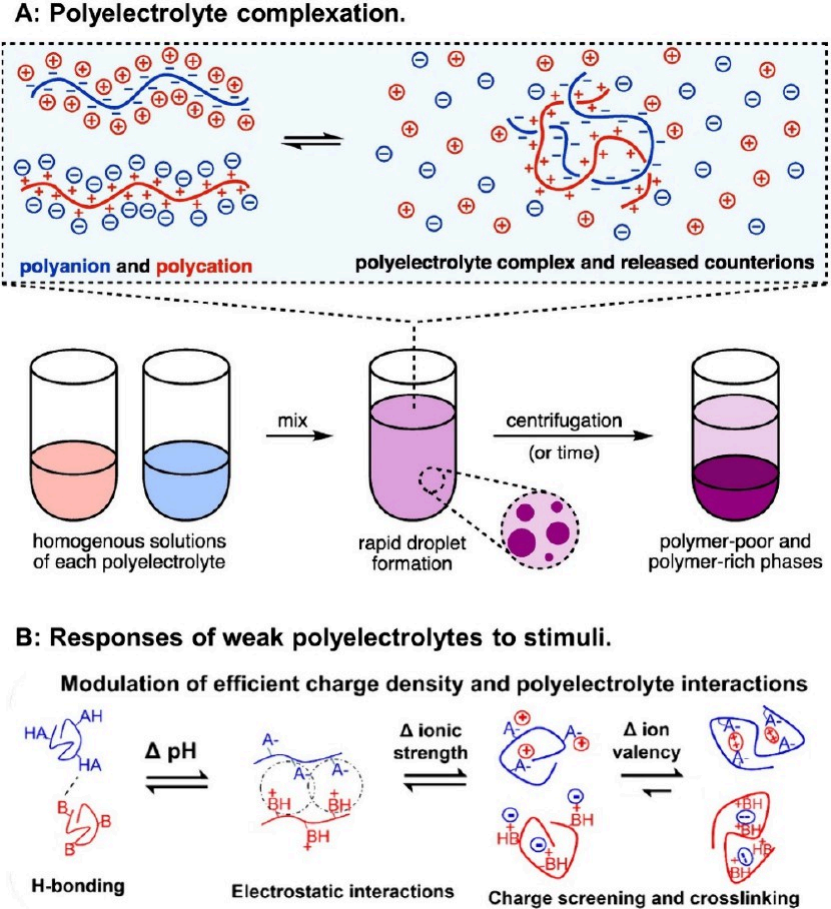
(A) Schematic of polyelectrolyte complexation; the resultant liquid–liquid phase separation is depicted on both microscopic and macroscopic levels. Reproduced with permission from ref (143). Copyright 2021 Elsevier. (B) Responses of weak polyelectrolytes to stimuli. Schematic depiction of the molecular and conformational responses of weak polyelectrolytes bearing either weak acid (AH) or basic (B) moieties with respect to pH and salt stimuli. Reprinted with permission under a Creative Commons CC_BY 4.0 license from ref (144). Published 2022 MDPI.

Polyelectrolytes are often characterized by charge properties depending on pH, and these variations significantly affect their applications. They are classified as “strong” if their charge remains constant over the entire pH range (1–14), while “weak” polyelectrolytes have pH-dependent charges. This allows for a reactive character and is therefore more relevant in the field of nanomedicine. It should be also noted that, in the case of polyelectrolytes, the apparent p*K*_a_ value is more common, in contrast to the intrinsic p*K*_a_ value of a single molecule which rather considers the ratio of all ionized to deionized groups of a whole structure or composition, e.g., in a nanoparticle, hydrogel, or assembly.^137^ For polyelectrolytes, the apparent p*K*_a_ values indicate the pH at which half of the repeating monomeric units are charged. The apparent p*K*_a_ is influenced by the substituents on the ionic group and the polymer composition (i.e., comonomers). Previously, Armes and co-workers demonstrated this phenomenon by titrating different polycations consisting of side-chain tertiary amines as ionizable groups.^138^ It was shown that the p*K*_a_ values of the tested amines greatly depended on their substitution (e.g., methyl vs ethyl) as well as the comonomer composition when assessing copolymers with different chargeable groups. They can furthermore be influenced by molar mass, conformation, counterions, and complexation.^139^ At high degrees of ionization, polyelectrolytes tend to exhibit extended chain conformations, while low degrees of ionization favor more compact structures.^140^ Changing the valence of the counterions from monovalent to multivalent (e.g., by mixing of polyelectrolytes with opposite charges) induces intramolecular electrostatic cross-linking,^141^ leading to additional conformational transitions (Figure 15). The electrical charges on polyelectrolytes are neutralized by the surrounding counterions, which are present in solvent, media, buffers, or physiological fluids. This leads to a significant decrease in the osmotic activity of the polyelectrolytes. This aspect is generally poorly investigated yet plays a crucial role. For example, highly charged polyelectrolytes neutralized by monovalent counterions exhibit only 15 to 20% of the ideal osmotic pressure. For polyvalent counterions, the reduction in osmotic pressure is even more pronounced, reaching only 1 to 3% of the ideal value.^142^ It is therefore of great importance to select an appropriate formulation method and solvent. Polyelectrolytes can interact through electrostatic interactions to form polyelectrolyte complexes (PECs). These complexes are formed when oppositely charged polyelectrolytes interact and reach a state of electroneutrality, which can lead to the release of counterions and precipitation of the complex.^143^ At elevated salt concentrations, these interactions are suppressed due to the screening of the polymer charges by the salts.

Although polyelectrolytes are known to be highly soluble in water, there is a strong dependency of this solubility on the pH value and the ionic strength of the surrounding media. When considering the pH value only, polyanions are generally very hydrophilic above their apparent p*K*_a_ value, while polycations are hydrophilic below their apparent p*K*_a_ value. In the non-charged state these polyelectrolytes are often hydrophobic due to limited or prevented hydration.^145^ A polyelectrolyte’s behavior is influenced by a number of factors, including electrical forces, entropic considerations,^146^ and random movement of the polymer chains (Brownian motion). Electrostatic repulsion between similarly charged segments of the polymer can lead to an extended chain conformation, affecting the solution’s rheological properties.

De Breuck et al. have shown this behavior for amino-acid-derived polycarboxylates with varying hydrophobicity.^42^ In general, the p*K*_a_ values of polyelectrolytes strongly depend on the structural characteristics of the polymer itself as well as the polymer concentration or the salinity of the solution.^147^ For example, PAA (p*K*_a_ = 6.21) features a lower p*K*_a_ value than PMAA (p*K*_a_ = 7.03) at the same molar polymer concentration (*c* = 0.01 M),^147^ while side-chain branching of amino-acid-derived polyacids does not impact the derived p*K*_a_ value.^42^ Increasing salinity leads to a decrease of the p*K*_a_ value of polyacids (polyanions), while the p*K*_a_ value of polybases (polycations) decreases.^148^ Especially for applications in biological compartments, one needs to be aware of the change in properties due to varying salt concentrations of the surrounding ions (e.g., chloride ions). However, this feature also enables the design of tailored, pH-responsive materials.

Polycations used for biomedical applications often contain amino groups as the pH-responsive unit. Here, the p*K*_a_ varies depending on the degree and type of alkylation of the amine. For example, the p*K*_a_ of ammonia (p*K*_a_ = 9.26) increases upon ethylation (p*K*_a,ethylamine_ = 10.75; p*K*_a,diethylamine_ = 10.98; p*K*_a,triethylamine_ = 10.76). Trützschler et al. reported a similar trend for the p*K*_a_ values of poly(2-aminoethylmethacrylate)s with varying substitution patterns (p*K*_a,poly(2-aminoethylmethacrylate)_ = 8.19; p*K*_a,poly(2-methylaminoethyl-methacrylate)_ = 8.40; p*K*_a,PDMAEMA_ = 7.45).^149^ These properties are crucial to the biocompatibility and performance of polycations, which will be discussed in section 5 below.

#### 4.2.1 Polyzwitterions

**Figure 16:**
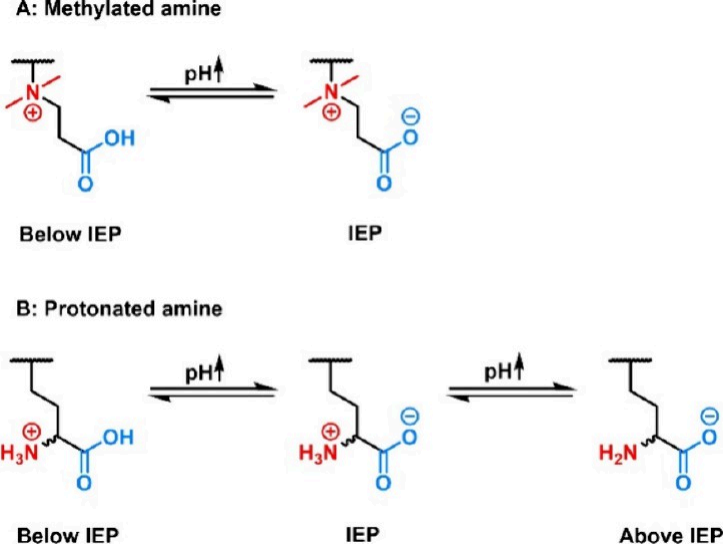
Impact of pH changes on the charge of different types of ZIPs. (A) ZIPs with methylated amines (polybetaines), exemplarily shown for PCBs. (B) ZIPs with protonated amines, exemplarily shown for polymers derived from α-amino acids.

Polyzwitterions are a special case as they contain both cationic and anionic groups. Compared to those of polycations and polyanions, the pH responses of polyzwitterions are more complex, as one needs to consider both anionic and cationic groups, which usually coexist in proximity and, thus, influence each other. At their isoelectric point (IEP), polyzwitterions are in a neutral charge state and exhibit the greatest hydrophobic character.^115,150^ This can result in precipitation of the polymers in aqueous solution.^122^ Above and below their IEP, polyzwitterions are of either anionic or cationic nature, respectively (Figure 16A).^150^

In this context, polybetaines—those polymers with a permanent positive charge, derived from their methylated amine—feature a relatively high pH range in which they are in the zwitterionic state. The range further depends on the nature of the anionic moiety. For example, carboxylates are weaker acids than sulfates or phosphates, leading to a decreased range of isoelectric state.^1^ Amino-acid-derived polyzwitterions are composed of carboxylates, which are weak acids, and primary amines, which are weak bases. Consequently, they are particularly susceptible to changes in pH (Figure 16B), leading to a very narrow IEP as well as tremendous pH-derived changes in properties, such as solubility and overall charge.^122^ For amino-acid-derived polyzwitterions, IEPs ranging from pH ≈ 4.5 to pH ≈ 6.5 were described. Interestingly, the IEP of different polymers was strongly influenced by structural alterations, such as the backbone chemistry of the tested polymers.^122,151^ Unfortunately, there have been few systematic studies of this to date. These studies are urgently needed in the future to truly understand the driving forces of polyelectrolyte properties and interactions with biological matter, such as proteins or membranes.

### 4.3 Characterization of Polyelectrolytes

Analysis of polyelectrolytes, including their size, charge, structure, and functionalization, is of vital importance to draw comprehensive information about structure–property relationships. The application of state-of-the-art techniques, such as size-exclusion chromatography (SEC), nuclear magnetic resonance spectroscopy (NMR), dynamic light scattering (DLS), and zeta potential measurements, is essential for the characterization of polymers including polyelectrolytes.^127,152^ To gain insight into the dynamic behavior of polyelectrolytes in biological environments, including their degradation, interaction with cells, and intracellular trafficking, sophisticated analytical methods are necessary.^153^ The following paragraphs aim to provide a short overview of analytical methods suitable for the characterization of polyelectrolytes, pointing out their potential and challenges. When applied to polyelectrolytes, these techniques present specific challenges, primarily due to the charged nature of polyelectrolytes and their interactions with the environment.

PECs formed by polyanions and polycations have attracted considerable interest due to their diverse and promising applications. To effectively study these complexes, a variety of advanced characterization techniques are employed. These methods, thoroughly reviewed by J. L. Lutkenhaus and colleagues,^154^ provide critical insights into the structure, dynamics, and interactions of polyelectrolytes, paving the way for optimizing their use in fields such as drug delivery, gene therapy, and materials science.

#### 4.3.1 Molar Mass and Composition

To determine the molar mass and its distribution in polyelectrolytes, SEC is commonly employed. One challenge is the interaction of polyelectrolytes with the column material, which can lead to adsorption and affect the accuracy of the molecular weight determination. Thus, polyelectrolytes containing protecting groups are used or water-based SEC is employed.^155^ Dynamic as well as static light scattering (DLS, SLS) can be also applied to measure the size distribution and polydispersity of large polyelectrolytes in solution.^156^ However, the strong scattering signals from polyelectrolytes due to their charge can complicate data interpretation. In addition, analytical ultracentrifugation (AUC) is employed to analyze the size, shape, and interactions of polyelectrolytes without the need for a stationary phase, reducing the risk of interactions that could influence results.^157^ Since polyelectrolytes can form complex aggregates or have different conformations, which, in turn, affect other parameters, interpretation of the results is not straightforward. NMR provides detailed information about the degree of polymerization, chemical structure, composition, and molecular dynamics of polyelectrolytes.^158^ However, the presence of charged groups can result in broadening of NMR signals, making it difficult to resolve detailed structural information. Fourier transform infrared spectroscopy (FTIR) is used to identify functional groups and study chemical bonding and molecular interactions.^159^ One challenge for the characterization of polyelectrolytes is that the hydration layer can interfere with the FTIR absorption spectra.

#### 4.3.2 Charge and Ionization

In the case of polyelectrolytes, the apparent p*K*_a_ plays a crucial role^160^ linked to other parameters, e.g., charge and size. Zeta potential measurements via electrophoretic light scattering (ELS) are employed to determine the surface charge and stability of polyelectrolyte solutions or colloidal suspensions.^161^ Here, the influences of the ionic strength and pH value of the medium should be considered, which can significantly alter the zeta potential. Potentiometric and conductometric titrations can be used to investigate the ionization behavior and charge density of polyelectrolytes in solution but require large sample amounts. Thus, polyelectrolyte-assisted charge titration spectrometry (PACTS) can be applied for materials showing phase separation and light scattering signals.^162^ In general, high ionic strength and the presence of multivalent ions can complicate the titration curves and make accurate determination and lab-to-lab comparisons difficult.

#### 4.3.3 Morphology

As polyelectrolytes are often utilized as nanoparticles or used for other assembled structures, their morphology and surface characterization can be characterized by various microscopy techniques.^163,164^ Scanning electron microscopy (SEM) provides high-resolution images of the surface morphology, but drying and coating with conductive materials can alter the “true” morphology. Transmission electron microscopy (TEM) offers detailed images of the internal structure, but sample preparation can be challenging due to the sensitivity to electron beam damage. SEM is fast and relatively unrestricted and can be performed with minimal sample preparation. However, when polymers need to be analyzed in detail, TEM is used. Atomic force microscopy (AFM) is used to study surface topography and mechanical properties at the nanoscale, but the soft and hydrated nature of polyelectrolytes can lead to artifacts in the measurements. X-ray diffraction (XRD) offers insights into the crystalline structure of solid polyelectrolytes;^165^ however, polyelectrolytes may exhibit poor crystallinity, making it difficult to obtain clear diffraction patterns.

**Figure 17:**
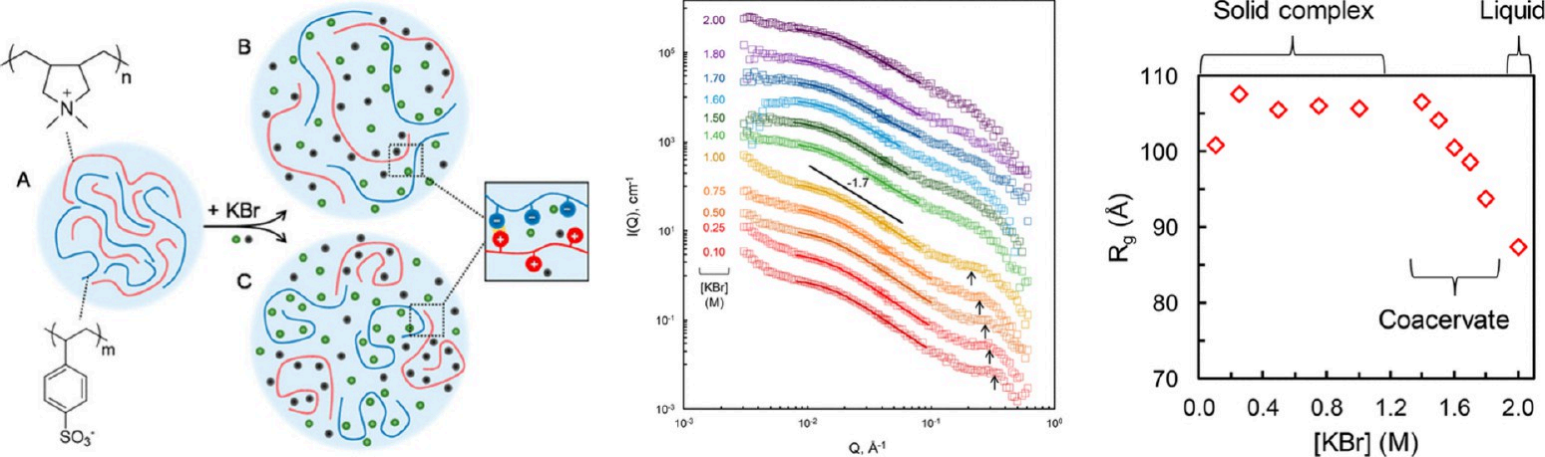
(Left) Schematic representation of the polyelectrolyte complex of the polycation/polyanion pair poly(diallyldimethylammonium) (PDADMA)/poly(styrene-sulfonate) (PSS) (A) and the same complex during the transition to the coacervate phase by addition of KBr as salt. Chains in the network are hypothesized to either (B) expand while remaining connected at certain polymer/polymer points, with salt either closely associated with the polyelectrolyte or in the surrounding solution, or (C) phase-separate into denser regions to maintain some polymer connectivity while remaining more tightly coiled, with excess salt and water occupying an adjacent microphase. (Middle) Small-angle neutron scattering profiles in polyelectrolyte complex samples with 0.1–1.0 M KBr. (Right) Radius of gyration (R_g_) resulting from fits of the form factor, P(Q), using the Debye function for Gaussian chains. Reproduced with permission from ref (168). Copyright 2018 American Chemical Society.

To gain insights into the fine structure of polyelectrolytes and in particular polyelectrolyte complexes (PECs), small-angle neutron and X-ray scattering (SANS, SAXS) have been employed.^166,167^ SAXS measures the scattering of X-rays at small angles, providing insights into the internal structure, such as the presence of microphases and aggregates and the organization of polyelectrolyte chains in solution. These techniques allow researchers to examine how the dimensions and structure of PECs respond to changes in the ionic environment, particularly the salt concentration. A study by J. B. Schlenoff and co-workers investigated observed changes in the coil dimensions of polyelectrolytes in response to increasing salt concentration (Figure 17).^168^ SANS profiles and radius of gyration (R_g_) measurements were applied for PEC samples across different salt concentrations. At a low concentration of 0.1 M KBr, the PEC is in a solid state, while increasing the salt concentration to 2.0 M KBr transforms the PEC into a liquid phase, passing through a series of increasingly fluid coacervate states between 1.4 and 1.8 M KBr.

Each of these techniques has its own strengths and weaknesses, and the techniques can be complementary when used together to provide a comprehensive understanding of the morphology and structure of polyelectrolyte assemblies. SAXS, for instance, is excellent for analyzing the bulk structure in solution, while techniques such as EM, AFM, and XRD can provide more specific information about the surface, crystallinity, and other structural features.

#### 4.3.4 Thermal Characterization

Differential scanning calorimetry (DSC) measures thermal transitions such as glass transition temperature, melting point, and crystallization behavior.^169^ Thermogravimetric analysis (TGA) determines thermal stability and composition by measuring weight changes under controlled temperature conditions.^170^ The high affinity of polyelectrolytes for water can lead to difficulties in the interpretation of thermal transitions, since the dehydration and decomposition of polyelectrolytes often overlap.^171,172^ In addition to pH, the temperature can also affect the conformation and solubility of polyelectrolytes. Determining their lower critical solution temperature (LCST) involves evaluating their thermal response, typically using techniques such as turbidimetry to determine the precise temperature and concentration at which phase separation occurs.^173^ Understanding the LCST of polyelectrolytes is critical for applications that require temperature-sensitive behavior, such as controlled drug release and smart hydrogel formation.

#### 4.3.5 Rheological and Mechanical Properties

The flow and deformation behavior of polyelectrolytes are determined by rheometry.^174^ The challenge is the shear sensitivity of polyelectrolytes and their tendency to gel. Tensile and compression tests evaluate the mechanical strength and elasticity of polyelectrolyte films and gels, relevant for biomedical applications in the field of tissue engineering.^175^ Polyelectrolytes can be highly sensitive to humidity and environmental conditions, which can influence their mechanical properties during testing. Viscosity measurements of polyelectrolytes can also play a crucial role in manufacturing technology. They are essential for process optimization because viscosity affects the processability of polymers in various manufacturing processes such as extrusion, injection molding, and coating. The viscosity of polymers is measured by methods such as capillary viscometry, rotational viscometry, falling ball viscometry, and rheometry.^174,176^ Each method has its specific applications and advantages, depending on the type of polymer and the measurement conditions. Factors influencing the viscosity of polymers include molar mass, temperature, degree of branching, and the solvent used. A higher molar mass generally leads to a higher viscosity, while highly branched polymers can have a lower viscosity compared with linear polymers of the same molecular weight. However, the viscosity of polyelectrolytes is also influenced by the surrounding medium and counterions, as these affect the interactions between the polymer molecules. Compared to apolar and non-ionic polymers, polyelectrolytes often feature a counterintuitive viscosity in solution.^177^ In particular, the die relative viscosity of a semiflexible polyelectrolyte increases during the dilution process. This inverse behavior is caused by intermolecular charge-mediated repulsions and was, for example, demonstrated for quarternized poly(*N*-butyl-4-vinylpyridinium bromide).^178^

#### 4.3.6 Imaging

For studying biological interactions, confocal laser scanning microscopy (CLSM) is often employed, which allows the visualization of polyelectrolyte interactions with cells and tissues. The challenge here is the need for appropriate fluorescent labeling without alteration of the polyelectrolyte’s properties. Spatio-temporal image correlation spectroscopy and super-resolution microscopy provide a deeper understanding of intracellular delivery of polymers.^179,180^ Flow cytometry is used to study the binding and uptake of polyelectrolytes by cells.^121,122^ The aggregation tendencies of polyelectrolytes lead to particular challenges in accurate data interpretation, as the fluorescence signal itself can be altered.

## 5 Cellular Interactions

### 5.1 General Considerations of Charged Polymers for Biological Applications

As mentioned above, the interactions between polyelectrolytes and the surrounding media play important roles in biological applications, since osmolarity, salt concentration, and pH affect the polyelectrolytes, which can be considered as an advantage (trigger) or an obstacle (stability). These characteristics are especially valuable in targeted drug delivery, where the tunable charge of polyelectrolytes enables them to respond to specific environmental triggers, facilitating controlled drug release. In coating applications, polyelectrolytes offer anti-bacterial and anti-fouling properties, which are critical for surfaces exposed to microbial environments or prone to biofouling. Here, their charged nature and ability to form a polarizable electrical double layer enhance the polyelectrolytes’ binding and repellent capacities, providing protection against unwanted adsorption of microorganisms or contaminants.^181^

By integrating further interactions, e.g., with hydrophobic moieties, the electrostatic interactions can be complemented by hydrophobic interactions, which positively influence the colloidal stability, making such polyelectrolytes ideal candidates for applications requiring stable nanocarriers.^182^ This property is widely exploited in nanomedicine, where stable nanocarriers are essential for effective drug encapsulation, protection, and targeted delivery within biological systems; delivery of genetic material, such as DNA and RNA, is one of the well-known applications.

Thus, polyelectrolytes with pH-dependent functional groups warrant particular attention. They can alter their charge states depending on the environmental pH, influencing solubility, molecular conformation, and ultimately their interactions with biological targets. This pH-dependent tunability allows for site-specific drug release and tailored interactions within diverse physiological conditions, making such polyelectrolytes highly attractive for applications requiring precise control over molecular configuration and bioavailability.

Thus, polyelectrolytes are highly versatile materials for diverse applications, from drug delivery and anti-bacterial coatings to anti-fouling surfaces. The following section highlights the properties of polyelectrolytes for nanomedicine and shows how polyelectrolytes can be used to achieve effects beyond those of single charges. By leveraging their multifunctional capabilities, polyelectrolytes can effectively mediate cellular interactions, enhance therapeutic efficacy, and introduce new possibilities in the development of next-generation responsive materials.

### 5.2 Challenges of Polyelectrolytes for Biological Applications

**Figure 18:**
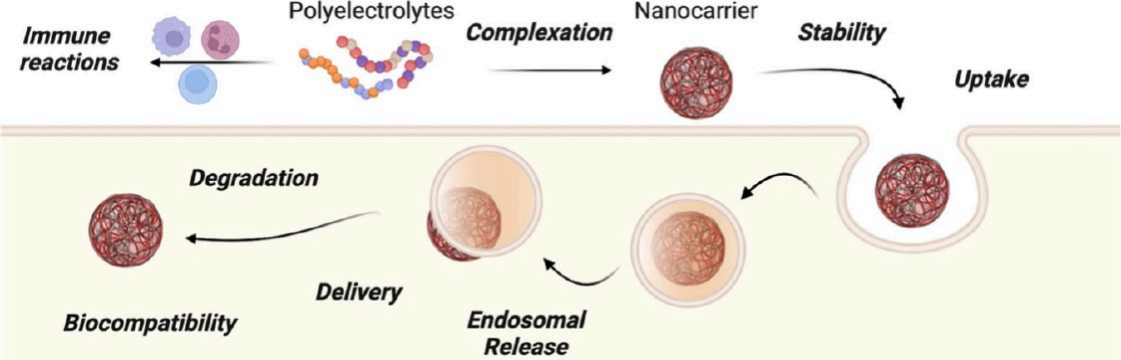
Overview of hurdles (highlighted in bold) associated within the use of polyelectrolytes in biological applications.

Polyelectrolytes present a multitude of challenges in the field of nanomedicine (Figure 18), largely due to their responsiveness and interactions within biological systems. Achieving precise control over the molar mass and charge density of polyelectrolytes is of paramount importance for maintaining consistent biological activity. Furthermore, the functionalization of polyelectrolytes with targeting ligands or other functional groups represents a significant challenge, as off-target effects cannot be excluded because polyelectrolytes contain multiple charges, complicating ligand–receptor interactions. Furthermore, polyelectrolytes have the potential to non-specifically bind to proteins, cells, and other biological molecules,^33,183^ which may result in reduced targeting efficiency and the possibility of adverse effects.

First, the potential for biocompatibility and toxicity must be considered, as a high charge density, particularly in polycations, has the potential to disrupt cell membranes and induce cytotoxicity. Thus, an optimal balance between the envisioned biological effect, e.g., association, delivery, or uptake, and biocompatibility needs to be achieved.^184^ Furthermore, polyelectrolytes have the potential to elicit an immune response, which may result in immune activation or rapid clearance from the body,^185,186^ representing a challenge in the design of polyelectrolytes for nanomedical applications.

In physiological conditions, polyelectrolytes tend to complex, aggregate, or precipitate, which can affect their delivery efficiency and bioavailability.^187^

Due to the challenges with polyelectrolytes in a biological environment, the following section will focus on the specific effects and applications of polycations, polyanions, polyzwitterions, and polyampholytes. Finally, we highlight well-known representatives and their distinct applications.

### 5.3 Polycations and Their Distinct Features

**Figure 19:**
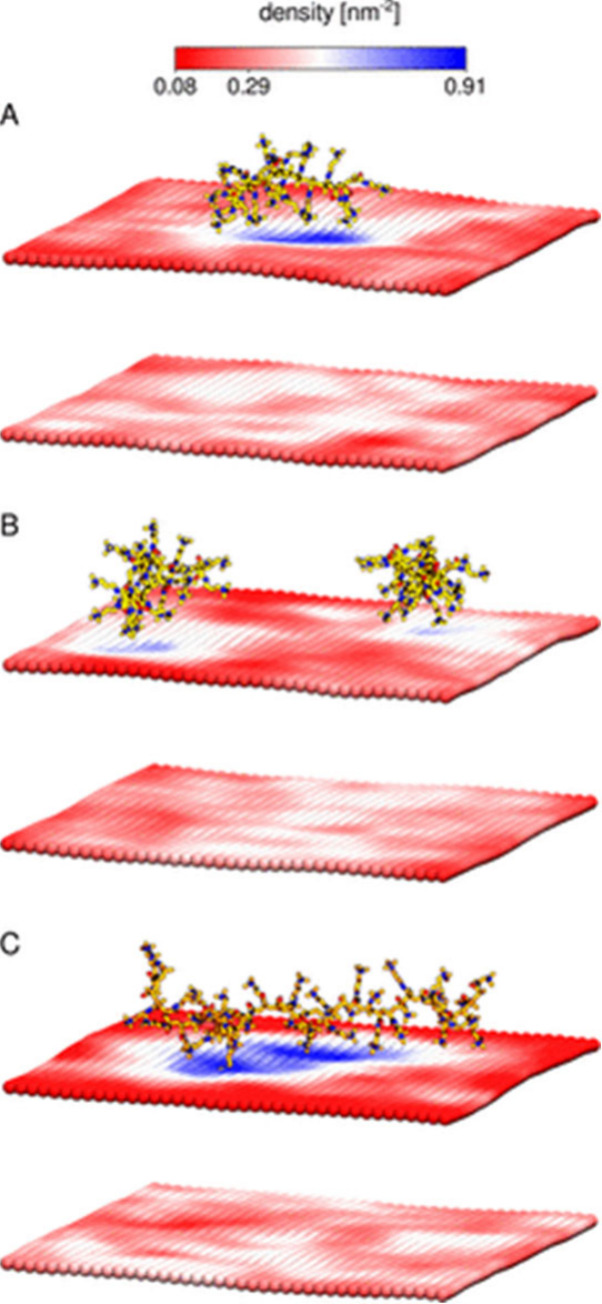
Two-dimensional density profiles of model membranes containing two lipids: zwitterionic 1-palmitoyl-2-oleoyl-*sn*-glycero-3-phosphocholine (POPC) and anionic 1-palmitoyl-2-oleoyl-*sn*-glycero-3-phosphoserine (POPS). The influence of the molar mass of the cationic polymer (poly([3-(methacryloylamino)propyl]trimethylammonium chloride) having 20 and 40 repeating units are shown: M20-1 (A), M20-2 (B), and M40-1 (C). Reprinted with permission under a Creative Commons CC_BY 4.0 license from ref (188). Published 2020 American Chemical Society.

The use of polycations in biological applications can be traced back to the mid-20th century when scientists first began exploring their potential in drug and gene delivery. The positive charges on the polycation attract negatively charged phospholipids and glycoproteins, enhancing the adhesion and uptake of polycations. Atomic-scale molecular dynamics simulations were used to demonstrate that, due to strong hydration, a polymer containing quaternized amines interacts with the phospholipid headgroup and not the acyl chain region. In addition, the adsorption of polycations on membranes leads to the formation of anionic, lipid-rich domains, an effect that increases with increasing degree of polymerization (Figure 19).^188^ This explains the known effect of molecular mass on the delivery and toxicity of polyelectrolyte carriers, with higher molecular mass polycations tending to be more effective but also more toxic,^189^ both due to interactions with cellular membranes. Thus, the degree of polymerization is critical for optimizing the balance between efficacy and safety in polyelectrolyte-based therapeutics.

**Figure 20:**
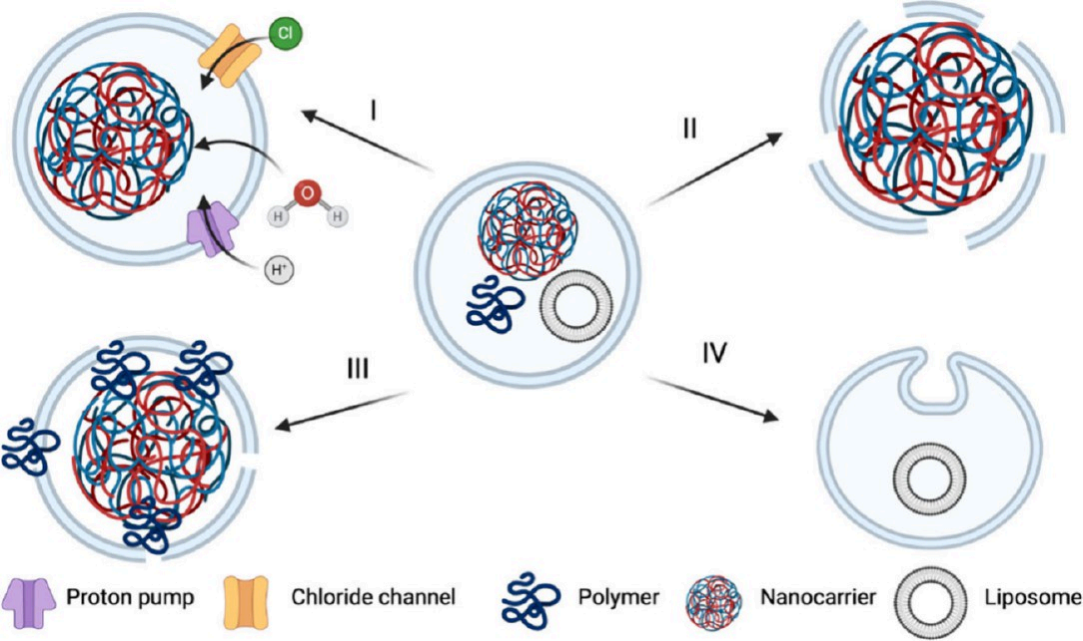
Overview of possible mechanisms for polymers crossing the endosomal membrane: I) osmotic rupture; II) polymer stretching; III) membrane destabilization; IV) membrane fusion.

Due to the polycation’s charge and size, its interaction with membranes results in its engulfment via endocytosis, whereby the polycations are transported into the cell within endosomes.^190^ As the polymers are unable to cross cellular membranes independently, the polycations must facilitate their release from endosomes, which represents a pivotal step in the successful delivery by polycations.^191^ The mechanisms by which polycations facilitate endosomal release remain a topic of active research.^192^ One well-established mechanism is the “proton sponge” effect.^193^ Many polycations contain pH-sensitive amines and have high buffering capacities. They act as “proton sponges” by neutralizing the acidic environment of the endosomes, which leads to an influx of ions and water (osmotic rupture, Figure 20). This influx causes the endosomes to swell and eventually burst, releasing the genetic material into the cytoplasm. Additionally, some polycations can directly disrupt the endosomal membrane by interacting with the membrane lipids, which also facilitates the release of genetic material into the cytoplasm (membrane destabilization).^194^ Polycations, in particular pH-responsive ones, are protonated due to the pH decrease of endosomal maturation. This leads to increased hydration and electrostatic repulsion and finally stretching of the polymer chains, leading to membrane rupture (polymer stretching). In the case of lipids or specifically modified polymers, fusion with the membrane can also lead to endosomal release, a mechanism also known from viruses (membrane fusion).

Following its release from the endosome, a polycation can also interact with microtubules, a cellular network for intracellular transport.^195,196^ Furthermore, interaction with the highly charged mitochondria membrane can also occur, a side effect due to the high charge density of some polycations.^197^

The capacity to deliver therapeutic genes to targeted cells is a cornerstone of gene therapy. In this context, polycation-based vectors play a pivotal role. Tailored polycations with the aim of boosting transfection efficiency, minimizing cytotoxicity, and targeting specific cell types have been presented.^198^ Polycations, due to their positive charge, interact with negatively charged nucleic acids (DNA and RNA, containing phosphates) through electrostatic interactions and are driven by entropy of the released counterions. The formation of polyplexes serves several critical functions. Primarily, polycation–nucleic acid complexes, also known as polyplexes, protect the genetic material against nuclease-mediated degradation. Second, the condensation of the genetic material by polycations results in more compact structures, which facilitates cellular uptake and improves the efficiency of transfection.^199^ The cationic charges also facilitate transportation across cellular membranes, a process known as “polyplex surfing”.^200−202^

The interactions of polycations and membranes can also be used for applications beyond gene delivery. Polycationic peptides like polymyxins were discovered in the 1940s and have been pivotal in combating infections caused by Gram-negative bacteria.^203^ Their ability to disrupt bacterial membranes and their resurgence as last-resort antibiotics underscore their historical and ongoing importance in medicine. Polycations show the potential to disrupt bacterial cell membranes due to their positive charge, making them effective against antibiotic-resistant bacteria. However, the composition of membranes, in particular their anionic lipids, plays a crucial role.^204^

It should be noted that interactions of polycations with cell membranes are carefully balanced to guarantee their efficacy while simultaneously minimizing the potential for cytotoxic effects such as membrane disruption and penetration. Accordingly, a variety of strategies have been proposed to circumvent this dilemma, e.g., PEGylation^205^ or complexation with polyanions.^206^ Furthermore, a moderate charge density can ensure effective binding and uptake without causing significant damage to the cell membrane.^207^ The use of low-molecular-weight polymers has been demonstrated to result in reduced cytotoxicity. An additional factor to consider is the balance between the hydrophilic and hydrophobic properties. Hydrophilic polymers are generally considered to be more biocompatible, as they interact less with cell membranes and proteins, thereby reducing unwanted immune reactions. Increasing hydrophobicity can, on the other side, also increase colloidal stability.^208^

#### 5.3.1 Prominent Examples: Synthetic, Non-degradable Polycations

**Figure 21:**
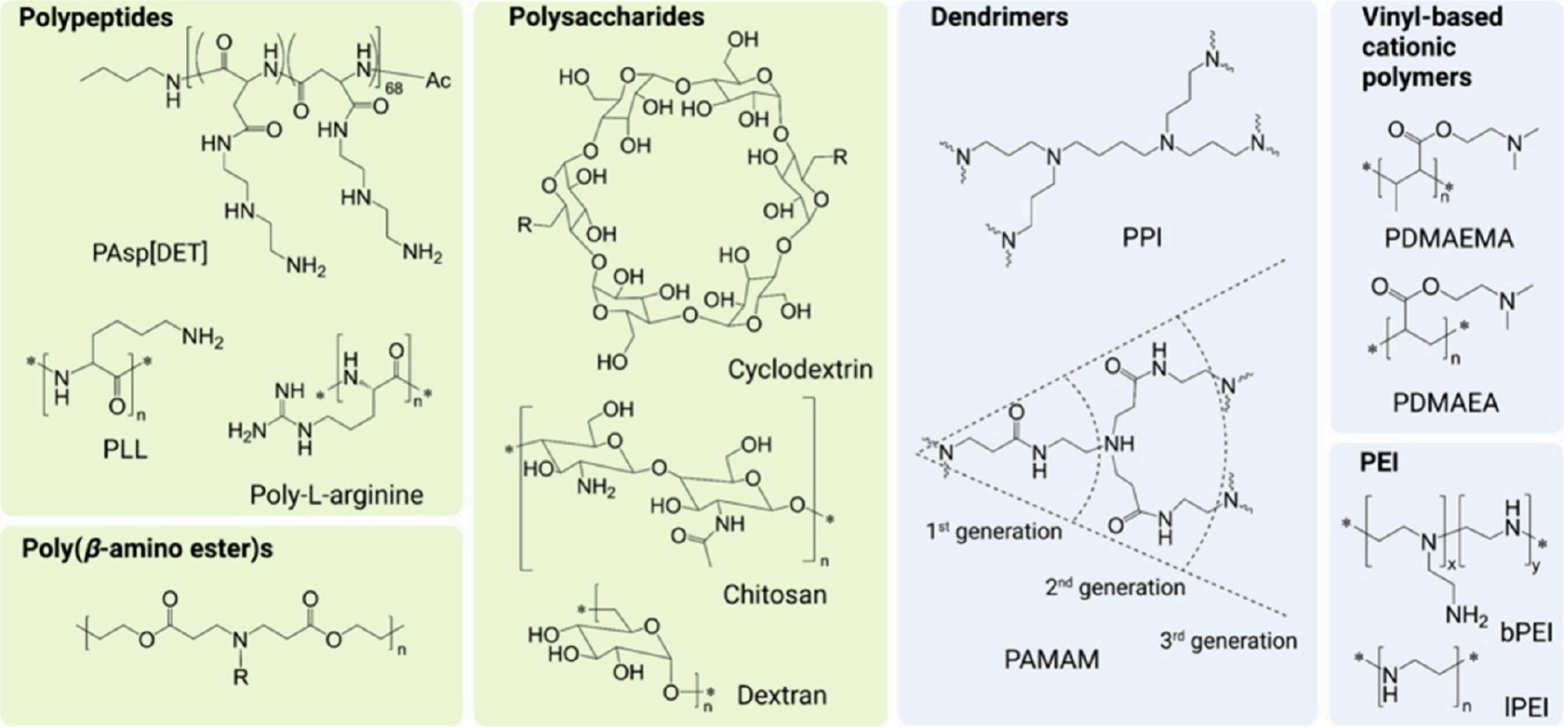
Chemical structures of selected polymeric vectors commonly used for gene delivery. Biodegradable polymers are highlighted in green, and non-biodegradable polymers are highlighted in blue. The * indicates the end group. R is a possible modification with functional amino groups. Reproduced with permission from ref (199). Copyright 2023 Springer.

PEI (Figure 21) is one of the oldest, commercially available, and therefore best-studied synthetic polycations. Its high cationic charge density allows for robust DNA binding and efficient endosomal release via the proton sponge effect. It has the highest charge density of amines, and in the case of b-PEI, primary, secondary, and tertiary amines are combined to enhance its binding efficiency. Another unique feature of PEI is that the amines are located in the backbone of the polymer, resulting in steric requirements that are different from those of polymers that carry side-chain charges. This unique structure contributes to PEI’s effectiveness in gene delivery applications. However, variants with high molar mass can be more cytotoxic, requiring careful optimization to balance efficacy and safety.^209^ Several approaches have been developed to circumvent the well-known efficacy/toxicity dilemma associated with PEI. Modifications such as the incorporation of cross-linkers^210^ or cleavable groups that degrade in response to specific stimuli and the addition of targeting moieties^211^ to direct the polymer to specific cells or tissues have shown promise. These strategies aim to enhance the therapeutic potential^60^ and delivery efficiency while reducing cytotoxicity and side effects.

PDMAEMA is the best-known representative of vinylic polycations and is frequently used to elucidate structure–property relationships due to its possibilities for controlled synthesis.^81,212^ PDMAEMA is a versatile cationic polymer that can be synthesized with precise control over its molecular weight, composition, and architecture, making it an ideal candidate for systematic studies on how polymer structure and architecture influence physical properties and biological interactions.^213−215^ Its pH- and temperature-dependent solubility enables reversible transitions between hydrophilic and hydrophobic states, which are useful in applications such as gene delivery. Its ability to be synthesized by controlled polymerization techniques further enhances its utility in fine-tuning polymer properties for specific applications.^216^

In addition to PDMAEMA, similar polymers with acrylamide backbones and various amine groups have also been studied extensively.^207,217,218^ These polymers share the beneficial feature of allowing systematic modifications of their chemical structure, allowing researchers to explore a wide range of functional properties, including anti-fouling and anti-bacterial properties as well as flocculation.^219,220^ This adaptability forms the basis for AI/ML-optimized methods, where large data sets generated from different polymer structures and their corresponding properties are used to develop predictive models.^109,172^ Such models can accelerate the design and optimization of new polymers with the desired characteristics, paving the way for advanced applications in biotechnology and medicine.

#### 5.3.2 Synthetic, (Bio)degradable Polycations

Biodegradable polycations can be degraded or cleaved at their backbone and can therefore often be broken down into the smallest units, offering different advantages and challenges. The primary challenge for degradable polycations is to balance their degradation rate with their therapeutic efficacy. While their biodegradability reduces long-term toxicity and allows for a more controlled release of therapeutic agents, it also means that their functional lifetime in biological systems is limited.

**Poly(beta amino esters) (PBAEs)** are an interesting class of cationic but also hydrophobic and biologically degradable polyesters, described in the 1980s^221^ and first introduced by the R. Langer lab for gene delivery in 2000.^222,223^ These polymers have shown the ability to function for gene delivery and can act as immunostimulants.^224^ However, as PBAEs degrade to lower molecular weight fragments and free polymers, their immunostimulatory effects are significantly reduced. Due to the flexible synthesis methods, different architectures and functionalities can be synthesized, showing different performance, stabilities, and degradation and targeting possibilities.^225,226^ Currently, AI-based methods are also being employed to identify optimal polymer compositions.^227^ Due to the possibility of large material libraries, different structure–activity relationships have been identified. (i) An increase in hydrophobicity has been shown to improve the transfection efficiency of PBAE nanoparticles.^228^ (ii) The transfection efficiency of amine-terminated polymers is typically higher than that of acrylate-terminated polymers,^229^ which can be attributed to their higher positive charge and superior interaction with cellular membranes. (iii) The preference for linear PBAEs is based on the enhanced biocompatibility and the greater ability to control the size of nanoparticles, which is advantageous for scaling up the formulation and encapsulating a greater quantity of polynucleotides.^226^ However, the use of highly branched PBAEs is associated with enhanced stability and robustness.^230^ Despite PBAEs’ status as promising polycations, their implementation is hindered by regulatory constraints, thus the lack of experience with industrial application of these materials and a sufficient GMP production.^226^

**Figure 22:**
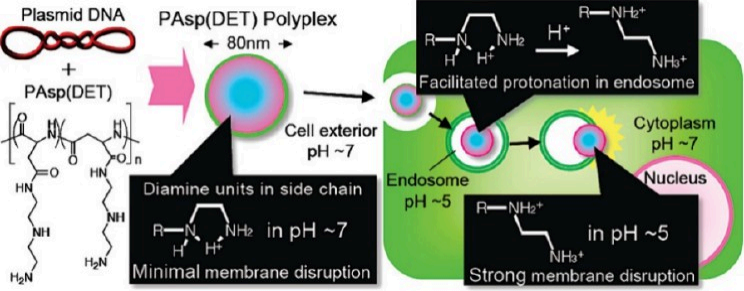
Chemical structure of a PEG-b-PAsp(DET) copolymer with a side-chain ethylenediamine moiety capable of forming stable polyplexes with pH-dependent protonation characteristics. This leads to an efficient translocation of polypeptides into cytosol. Reproduced with permission from ref (236). Copyright 2008 American Chemical Society.

**PAsp(DET)** is another biocompatible and pH-sensitive polycation, making it suitable for gene delivery with lower cytotoxicity compared to non-degradable polycations. It was introduced by the group of K. Kataoka^231^ to serve a specific pH-dependent conformational change (Figure 22).^92^ Detailed studies of PEI-inspired repeat units on the side chain provided further insights into the functionality and significance of protonation. It was shown that an even number of amines is conducive to transfection, due to increased buffer capacity and influenced by the neighboring protonation along a polymer chain.^232,233^ Recent progress was made by the incorporation of hydrophobic moieties that enhanced the delivery of mRNA, a rather amphiphilic nucleic acid.^234^ An increase in hydrophobicity results in enhanced stability yet concurrently diminishes the degradation of the particles.^235^ Therefore, a delicate equilibrium must be established.

Cyclodextrin-derived polycations, known for their cyclic structure, form stable inclusion complexes with hydrophobic molecules and exhibit lower cytotoxicity, making them promising for controlled release systems for various applications.^237^ M. E. Davis and co-workers introduced amine-functionalized cyclodextrin for gene delivery.^238,239^ The hydrophobic feature of cyclodextrin also enables codelivery of nucleic acids and small-molecule drugs.^240^

#### 5.3.3 Natural, Degradable Polycations

In addition to synthetic polycations, natural and degradable polycations offer significant advantages in terms of biocompatibility and reduced long-term toxicity. However, their application is often challenged by issues such as rapid degradation, variable physicochemical properties, and potential cytotoxicity at high concentrations. By employing strategies such as functionalization and coagulation, we are able to harness their full potential in biomedical applications.

**Poly-α-**l-lysine (PLL) and **poly-**l-arginine (PLR) are natural polycations used for gene delivery and surface modifications. These amino acids are also known from their presence in cell-penetrating peptides and histone proteins in nature, having distinct properties.^241^ Polymers using the alpha variant are biodegradable, and their cationic character facilitates strong interactions with negatively charged biomolecules such as DNA and cell membranes also due to their high p*K*_a_ and thus cationic character. However, their high apparent p*K*_a_ results in less-responsive behavior, limiting their endosomal release properties. To increase their endosomal release potential, functionalization with potent cationic moieties was applied.^242,243^ In addition, chloroquine and small endosomal enhancers^244^ were applied to increase their potential, even if rather for in vitro applications.

**Chitosan**, derived from chitin, or functionalized dextran, is a biodegradable polysaccharide used in drug and gene delivery systems due to its cationic functionalization. Besides its potential to deliver drugs, also its potential as a vaccine adjuvant was investigated.^245^ An interesting approach using polyelectrolyte nanogels was presented by L.-Q. Wang and co-workers.^246^ The pH-dependent cleavage of the side chain facilitated the release of the previously protected amines, whereby the polyanion exhibited a partial positive charge. Challenges with polysaccharides include their variable molecular weight, source, and degree of functionalization, which can affect their solubility and interaction with biological tissues due to batch-to-batch variability.

### 5.4 Polyanions and Their Features for Biological Applications

Polyanions have significant potential in various biological applications, particularly within in the field of nanomedicine.^247^ They generally exhibit lower cytotoxicity compared to polycations. This makes them more suitable for applications for which minimal cellular damage is critical. The negative charge of polyanions often results in electrostatic repulsion from negatively charged cellular membranes. Polyanions can encapsulate drugs, particularly positively charged or hydrophobic molecules, protecting them from degradation and facilitating controlled release.^248^ Polyanions can be part of polyelectrolyte complexes (PECs) that include both positive and negative charges, enhancing delivery systems; e.g., they can be used to coat viral and non-viral vectors, enhancing stability and targeting specific tissues while reducing unwanted side reactions.^248^ Recent studies showed that the direct interaction of this class of polymers can promote cell uptake in cancer cells.^249^ Polyanions can be part of systems that aid in the escape of therapeutic agents from endosomes,^250^ ensuring that the cargo reaches its intracellular target effectively.

#### 5.4.1 Heparins

One of the earliest and most well-known natural polyanions is **heparin**, an anti-coagulant drug that was discovered in 1917 and has been used in the clinic since 1935.^251^ Developed in the 1980s, low-molar-mass heparins represent a more sophisticated use of heparin as anti-coagulants. Their interactions with proteins involved in the clotting cascade illustrate the biological significance of polyanions. While heparin is effective, it can cause side effects such as heparin-induced thrombocytopenia.^252^

Some polyanions have shown potential in disrupting viral and bacterial adhesion to host cells, providing a mechanism for preventing infections.^253^ Another example, **poly(acrylic acid) (PAA)**, is used in various biomedical applications, including drug delivery and tissue engineering, due to its ability to form hydrogels and interact with biological molecules.^254^

#### 5.4.2 Polyglutamic Acid (PGA)

**PGA** is a biodegradable polyanion that can form complexes with drugs, enhancing their solubility and stability; its hydrophilic nature and biocompatibility make it an excellent carrier for drug delivery.^255^ Furthermore, it can be functionalized with antibodies, enabling a targeted delivery of nanocarriers.^256^ It can form nanoparticles and hydrogels that encapsulate drugs, protecting them from degradation and controlling their release. This controlled release is particularly beneficial for delivering chemotherapeutic agents, peptides, and proteins, ensuring that the drugs reach their target sites in the body at therapeutic concentrations over extended periods. PGA–drug conjugates enhance the solubility and stability of hydrophobic drugs, improving their bioavailability and reducing side effects.^257^ For instance, PGA–paclitaxel conjugates have shown promise in preclinical studies for their ability to target tumors more effectively than conventional formulations.^258^ In addition to its pharmaceutical applications, poly-γ-glutamic acid is also used in personal care products for its ability to retain many times its weight in water, improving skin texture and elasticity.^259^

#### 5.4.3 Eudragit S/L

**Eudragit** polymers have been widely used in the pharmaceutical industry for oral drug delivery, playing a crucial role in improving the efficacy and stability of medications. These polymers, developed by Röhm GmbH (now Evonik Industries), have a long history dating back to the 1950s.^106^ Eudragit polymers are a family of methacrylate-based copolymers, each designed with specific functional properties to address various challenges in drug delivery. The primary application of Eudragit polymers is in the formulation of tablet coatings, which protect the labile drugs from the acidic environment of the stomach. This protection ensures that the drug remains intact until it reaches the more neutral pH of the intestine, where it can be effectively absorbed. Eudragit S and L (containing PMAA as anionic moiety) are commonly used for targeted drug release to specific regions of the gastrointestinal tract.^260^ The ability to protect and control the release of drugs revolutionized the pharmaceutical industry, leading to the development of more effective and patient-friendly oral medications.

#### 5.4.4 Polypropyl(acrylic acid) (PPAA)

**PPAA** is a pH-responsive polymer that has garnered significant attention for its potential in facilitating endosomal escape, a crucial step in effective drug and gene delivery.^261^ Structurally, PPAA is an analogue of PAA where the propyl group is attached to the polymer backbone, resulting in a structure that responds dynamically to pH changes. This responsiveness is due to the presence of pH-dependent carboxylic acid groups. When delivery systems such as nanoparticles or polyplexes enter cells via endocytosis, they are encapsulated in endosomes, which naturally acidify as they mature, transitioning from early endosomes with a pH of around 6.5 to late endosomes and lysosomes with a pH of approximately 4.5–5.5. In the acidic environment of endosomes, the carboxylic acid groups in PPAA are protonated, altering the polymer’s hydrophilicity and causing it to adopt a more hydrophobic and compact conformation.^250^ This protonation allows PPAA to insert into the lipid bilayer of the endosomal membrane due to its increased hydrophobicity, disrupting the membrane’s integrity by creating pores or causing destabilization. Furthermore, this hydrophilic-to-hydrophobic transition can induce polymer aggregation, which also contributes to the disruption of the endosomal membrane.

### 5.5 Polyzwitterions

**Figure 23:**
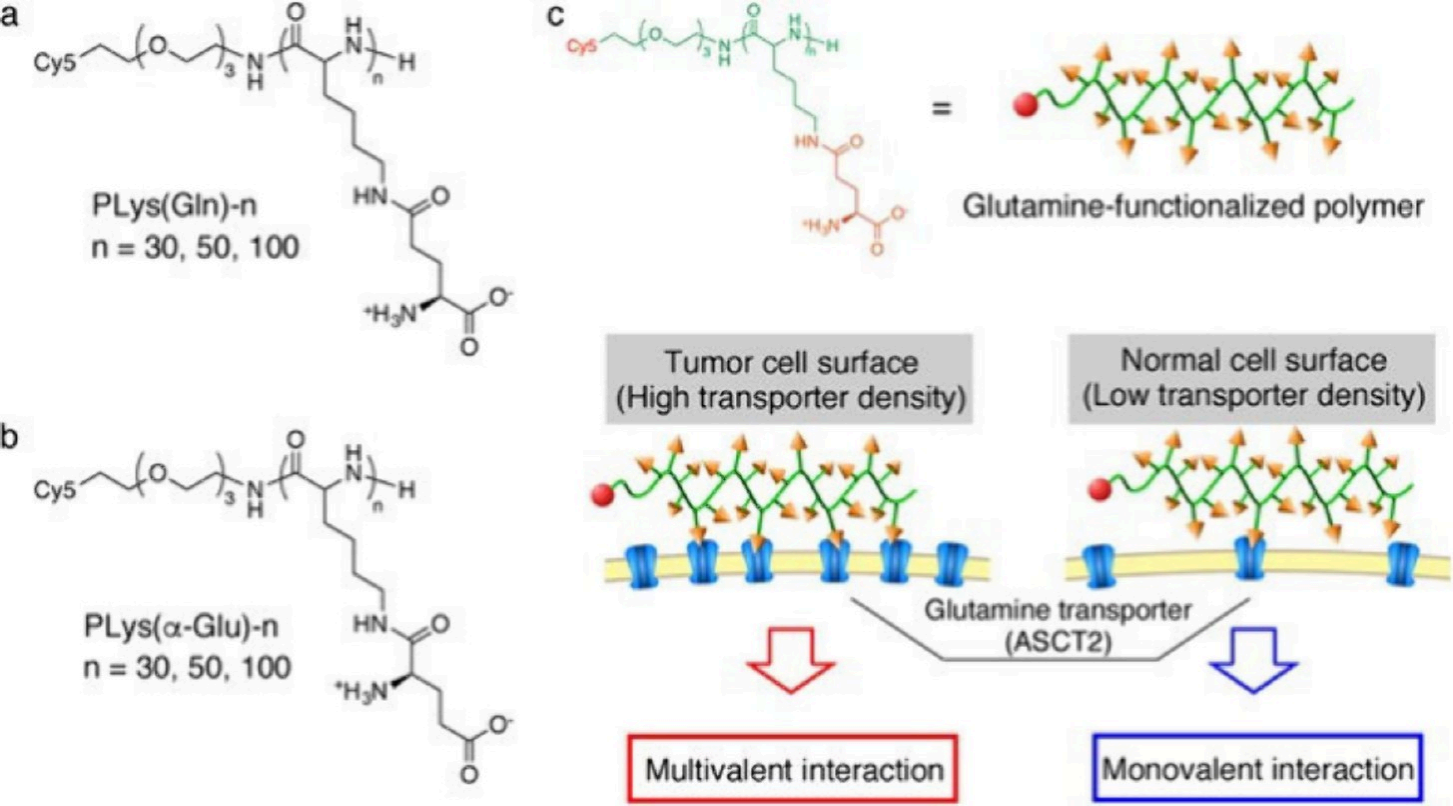
Design of a glutamine-functionalized polymer and interaction of the polymer with the cell surface. (a, b) Chemical structure of PLys(Gln)-n (a) and PLys(α-Glu)-n (b). (c) Illustration of the interaction between the glutamine-functionalized polymer and the cell surface. The polymer strongly interacts with tumor cell surfaces by multivalent interactions associated with high transporter density. In contrast, the polymer weakly interacts with normal cell surfaces because of low transporter density. Reprinted with permission under a Creative Commons CC_BY 4.0 license from ref (121). Published 2017 Springer Nature.

Among all polyelectrolytes, polyzwitterions and polyampholytes may be the most challenging to analyze when it comes to not only physicochemical properties but also driving forces for their interactions with biological matter. The properties of polyzwitterions in solution have been comprehensively reviewed by Blackman et al.^112^ Below or above their IEP, they may share some characteristics with polycations and polyanions, respectively.^122^ However, due to the existence of mixed charges over a broad range of physiological conditions, polyzwitterions share some properties with natural compounds, such as proteins or their building blocks, amino acids. These unique features render them interesting for applications in biological environments, e.g., for targeted drug delivery applications. In this context, cancer nanomedicine represents an important field, where tissue specificity would be beneficial to warrant site-specificity and reduce unwanted side effects. The exploitation of abnormalities in cancer cells compared to healthy, non-cancerous tissue via small, bioderived motifs appears to be a feasible strategy for targeting purposes.^262^ The fast cell growth of tumors requires a high demand of nutrients and consequently leads to overexpression of transporters such as glucose^263^ or AATs^264−266^ on the cell surface, which are readily available for interactions with polymers (Figure 23).^121^

**Figure 24:**
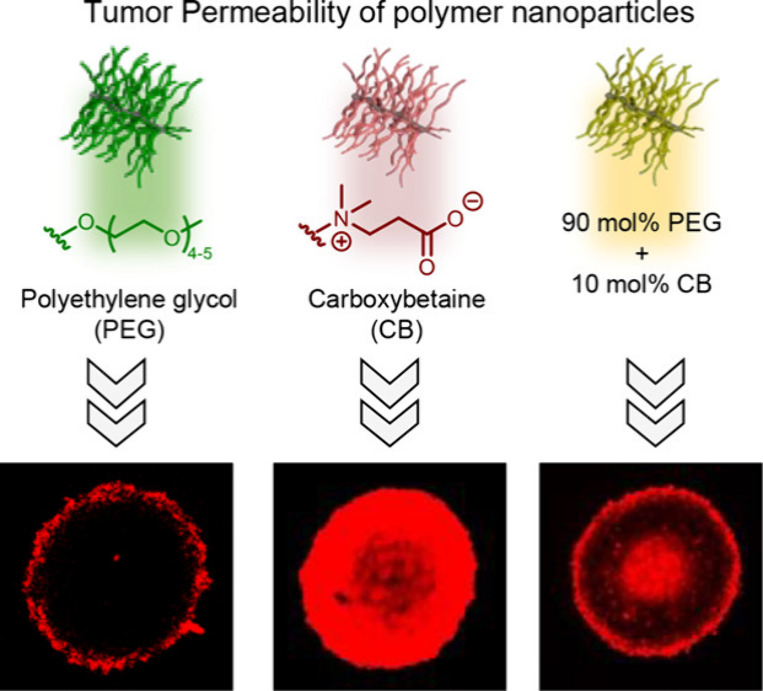
Diffusion of non-ionic and zwitterionic bottlebrush polymers into tumoroids. Reproduced with permission from ref (26). Copyright 2022 American Chemical Society.

In this context, polyzwitterions could have a major impact in modern oncology to deliver therapeutically active agents more safely and directly to the tumor.^26,267^ In particular for charged (zwitterionic) polymers, it has been assumed that these polymers can actively target cancer cells by interaction with AATs on the cell surface,^114,120−122,267^ rendering them as unique materials which may possess low-fouling characteristics combined with cellular specificity. Furthermore, the charged nature of polyzwitterions enhances their pH-dependent membrane interactions in a feasible manner, which can facilitate biocompatibility and intracellular endosomal release. In previous studies, increased accumulation of polybetaines in tumors was reported (Figure 24).^26,268^

It has further been assumed that this specificity is caused by specific interactions with cancer cells.^269^ More recently, the interaction of polyzwitterions with AATs has been proposed to be the origin of this specificity.^26,114,122^ While these indications are promising for future applications in the drug delivery area, there is still a serious lack of understanding of the specific interactions of polyzwitterions with cancer cells and AATs. Fujii and colleagues have shown that amino-acid-derived polyzwitterions are more specific than PCBs.^120^ Another study has shown that the involvement of AATs strongly depends on the properties of the tested polymers beyond the zwitterionic moiety.^122^ These results underline the urge for more systematic studies to truly understand the driving forces of the cell specificity of polyzwitterions in the future.

Due to their high solubility and potential to interact with different salts, zwitterionic polymers are also known for their stealthy behavior, which can be explained by a high polarization (Figure 13). The functionalization of polymers with cysteine through its thiol group represents a prominent example of their use in medical applications. This modification introduces amine and carboxyl groups at the polymer side chain or on surfaces, creating a superhydrophilic layer.^270,271^ Such surfaces are known for their stealth properties, which effectively allow them to evade immune detection, and exhibit excellent anti-fouling^270^ and anti-microbial characteristics,^271^ preventing the adhesion of unwanted biomolecules.^272^

### 5.6 Polyampholytes

In contrast to polyzwitterions, which contain both cationic and anionic groups within the same repeating unit, **polyampholytes** contain cationic and anionic groups on different repeating units. The potential to tune the ratio of cationic to anionic moieties renders them highly versatile for use in biological applications, where tailored charge balance and functionality are often critical.^113,273,274^ Their capacity to alter their electrical charge in response to fluctuations in environmental conditions, primarily pH levels, enables them to engage with a diverse array of biomolecules. This property is particularly advantageous in the context of drug delivery, gene therapy, and tissue engineering, where controlled interactions with cellular components are of paramount importance. The synthesis and analysis of large libraries of polyampholytes can be achieved by combining monomers of varying charges,^108,109^ thereby allowing the creation of unique materials with specific applications in mind. This combinatorial approach allows for the rapid development and optimization of materials with desired characteristics, such as enhanced biocompatibility, targeted delivery, and minimal toxicity.^83^

**Figure 25:**
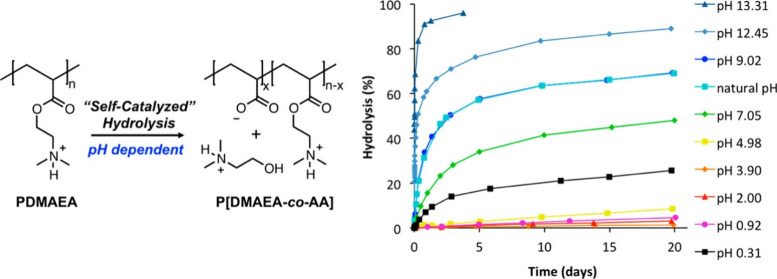
Mechanism and degree of pH-dependent hydrolysis of the charge-shifting polymer PDMAEA. Reproduced with permission from ref (278). Copyright 2020 American Chemical Society.

Additionally, **charge-shifting polymers**, which possess both cationic and anionic groups, offer the benefit of modulating their net charge in response to environmental stimuli.^275^ This feature can be employed to enhance the functionality and adaptability of advanced biomaterials for a range of biomedical applications, such as drug delivery systems.^84^ One prominent example is PDMAEA, the acrylate analogue to PDMAEMA, where the ester in the side chain can be cleaved under physiological conditions^85,276,277^ to give PAA and 2-dimethylaminoethanol (Figure 25).^278^ The hydrolysis process has been linked to the more efficient release of complexed double-stranded RNA (dsRNA) for incorporation into the RNA-induced silencing complex (RISC) and enhanced biocompatibility.^279^

## 6 Future Perspectives

During the past few decades, polyelectrolytes have emerged as promising materials for biological and biomedical applications. In particular, their enhanced hydration makes them attractive candidates to overcome issues of non-ionic polymeric equivalents. However, their charged nature and high density of functional groups often lead to difficulties in synthesis and characterization, which, in turn, further complicates the potential to draw adequate structure–property relationships. While established methods, such as the use of protecting groups, have been instrumental in improving the synthesis and stability of polyelectrolytes, recent advancements point to innovative synthetic strategies that could further enhance their applicability and functionality. Novel approaches, including orthogonal functionalization techniques and precision polymerization, allow for greater control over charge distribution and molecular architecture, potentially reducing synthesis-related limitations. To gain insight into the dynamic behavior of polyelectrolytes in biological environments, including their degradation, interaction with cells, and intracellular trafficking, sophisticated analytical methods and real-time monitoring are necessary. Advanced in situ imaging techniques, such as single-molecule tracking, high-resolution fluorescence microscopy, and time-resolved spectroscopy, can provide real-time monitoring of polyelectrolyte behavior in biological environments, including their interactions with cells and intracellular trafficking. These techniques offer more dynamic and accurate insights into the effects and side effects of polyelectrolytes in complex biological systems. Additionally, single-molecule fluorescence and fluorescence correlation spectroscopy (FCS) are emerging as powerful tools to track the behavior of individual polyelectrolyte chains in real time. These techniques can provide detailed information about dynamics, degradation processes, and interactions with biological targets. To address these challenges, interdisciplinary efforts are required, combining insights from chemistry, biology, materials science, and engineering to develop innovative solutions and advance the application of polyelectrolytes in nanomedicine.

The potential applications of polyelectrolytes in biomedical settings appear favorable, particularly considering the emergence of AI-based approaches to elucidate structure–property relationships. AI and ML algorithms can aid in processing large data sets from techniques like imaging, toxicity, SAXS, and AFM to identify patterns and predict the behavior of polyelectrolytes under varying conditions. This could help in designing more efficient polyelectrolyte-based materials by predicting their interactions with biological targets, their stability, and their performance in therapeutic applications. These enable researchers to anticipate the behavior of diverse polyelectrolyte structures within biological environments.

Additionally, balancing the delivery capacity and minimizing the cytotoxicity of cationic polyelectrolytes remain critical factors, especially for therapeutic applications. Although studies focus on optimizing charge density, molecular weight, and surface modifications to mitigate toxicity, more sophisticated strategies are needed to harness the potential of polyelectrolyte charges while minimizing adverse effects. For example, charges can be shielded by incorporating anionic groups or can be responsively modified to activate or deactivate under specific environmental conditions, thus enhancing biocompatibility and control over therapeutic interactions. Understanding the nature of their toxicity is essential for achieving safe and effective use of polyelectrolytes in biomedical applications, particularly in the targeted delivery of genetic material and other sensitive therapeutic agents.

Furthermore, the charged nature of polyelectrolytes can be exploited to create nanocarriers with specific interactions and stimuli responsiveness. Although the interaction of charges with the biological environment can present certain challenges, it also offers significant potential for the design of intelligent delivery systems. For instance, polyelectrolytes can be designed to interact with certain cellular receptors, thereby facilitating the targeted delivery of therapeutics. Furthermore, their responsiveness to environmental stimuli, such as pH and ionic strength, represents a driving force to facilitate the controlled release of drugs at the desired site of action. The ability to design nanocarriers with precise control over their interactions and responsiveness opens new possibilities for targeted and efficient therapeutic delivery, thereby making a significant contribution to the fight against various diseases.

## Abbreviations

AAT: amino acid transporter
ACPA: 4,4′-azobis(4-cyanopentanoic acid)
AFM: atomic force microscopy
AI: artificial intelligence
API: active pharmaceutical ingredient
ASCT2: alanine-serine-cysteine transporter
ATRP: atom-transfer radical polymerization
AUC: analytical ultracentrifuge
Boc: *tert*-butyloxycarbonyl
*b*-PEI: branched polyethyleneimine
*b*-PPI: branched polypropyleneimine
Cbz: benzyloxacarbonyl
CLSM: confocal laser scanning microscopy
CROP: cationic ring-opening polymerization
CTA: chain-transfer agent
Cy5: cyanine-5
DLS: dynamic light scattering
DNA: deoxyribonucleic acid
DSC: differential scanning calorimetry
ELS: electrophoretic light scattering
EPR: enhanced retention permeation
FRP: free radical polymerization
Fmoc: fluorenylmethoxycarbonyl
FTIR: Fourier-transform infrared spectroscopy
IEP: isoelectric point
IUPAC: International Union of Pure and Applied Chemistry
*l*-PEI: linear polyethyleneimine
*l*-PEI: linear polypropyleneimine
LAT1: large neutral amino acid transporter
Lys: lysine
ML: machine learning
NCA: *N*-carboxyanhydride
NMP: nitroxide-mediated polymerization
NMR: nuclear magnetic resonance spectroscopy
PAA: poly(acrylic acid)
PACTS: polyelectrolyte-assisted charge titration spectrometry
PAMAM: poly(amidoamine)
PBAE: poly(β-amino ester)
PCB: polycarboxybetaine
PDMAEA: poly(*N*,*N*-(dimethylamino)ethyl acrylate)
PDMAEMA: poly(dimethylaminoethyl dimethacrylate)
PEAA: poly(ethylacrylic acid)
PEC: polyelectrolyte complex
PEG: polyethylene glycol
PEI: polyethyleneimine
PGA: poly(glutamic acid)
PLL: poly(α-l-lysine)
PMAA: poly(methacrylic acid)
PPAA: poly(propylacrylic acid)
PPB: polyphosphobetaine
PPM: post-polymerization modification
PSB: polysulfobetaine
RAFT: reversible addition–fragmentation chain-transfer
RDRP: reversible deactivation radical polymerization
RNA: ribonucleic acid
RU: repeating unit
ROP: ring-opening polymerization
protecting group: size-exclusion chromatography
SEM: scanning electron microscopy
SLS: static light scattering
SPPS: solid-phase peptide synthesis
*t*Bu: *tert*-butyl
TEM: transmission electron microscopy
TGA: thermogravimetric analysis
xCT: cystine/glutamate transporter
XRD: X-ray diffraction
ZIP: zwitterionic polymer
